# Sex Differences in Itch Perception and Modulation by Distraction – an fMRI Pilot Study in Healthy Volunteers

**DOI:** 10.1371/journal.pone.0079123

**Published:** 2013-11-18

**Authors:** Astrid Stumpf, Markus Burgmer, Gudrun Schneider, Gereon Heuft, Martin Schmelz, Ngoc Quan Phan, Sonja Ständer, Bettina Pfleiderer

**Affiliations:** 1 Department of Psychosomatics and Psychotherapy, University of Muenster, Muenster, Germany; 2 Department of Clinical Radiology, University of Muenster, Muenster, Germany; 3 Competence Center Chronic Pruritus, Department of Dermatology, University of Muenster, Muenster, Germany; 4 Department of Anesthesiology and Intensive care Medicine, Karl Feuerstein Professorship, Medical Faculty Mannheim, University of Heidelberg, Mannheim, Germany; National Research & Technology Council, Argentina

## Abstract

**Background:**

Even though itch is a common syndrome of many diseases there is only little knowledge about sex and gender differences in pruritus, especially in central itch perception and modulation. To our knowledge, this is the first fMRI study examining sex differences in perception and its modulation by distraction.

**Methods:**

Experimental itch was induced by application of histamine (0.1 mM) via microdialysis fibers twice at the left forearm and twice at the left lower leg in 33 healthy volunteers (17 females, 16 males). The brain activation patterns were assessed by fMRI during itch without and with distraction (Stroop task). Between the various conditions, subjects were asked to rate itch intensity, desire to scratch and pain intensity. In a second experiment in 10 of the 33 volunteers histamine was replaced by saline solution to serve as control for the ‘Stroop’ condition.

**Results:**

Women generally presented higher itch intensities compared to men during itch over the course of the experiment. A more specific analysis revealed higher itch intensities and desire to scratch in women during experimental induced itch that can be reduced by distraction at the lower legs when itch is followed by ‘Stroop’. In contrast, men depicted significant reduction of ‘itch’ by ‘Stroop’ at the forearms. Women depicted higher brain activation of structures responsible for integration of sensory, affective information and motor integration/planning during ‘itch’ and ‘Stroop’ condition when compared to men. No sex differences were seen in the saline control condition.

**Conclusion:**

Women and men exhibited localisation dependent differences in their itch perception with women presenting higher itch intensities and desire to scratch. Our findings parallel clinical observations of women reporting higher itch intensities depending on itch localisation and suffering more from itch as compared to men.

## Introduction

Itch is a very common symptom of many dermatological diseases. Especially chronic itch reduces quality of life and might lead to depression and anxiety symptomatology [1]–[3]. Sex and gender are increasingly perceived as important factors influencing the extent of symptomatology, treatment response and outcome [4]–[9].

Still there is only very little knowledge about sex and gender differences in chronic pruritus. Ständer et al. [10] examined a large sample of patients with chronic pruritus. In this study, females reported higher itch intensities and were more negatively affected by pruritus. In line with that Holm et al. [11] found that women were more affected by visible areas of atopic dermatitis than men. Uttjek et al. [12] could show that women suffering from psoriasis had different expectations related to dermatological care than male patients.

During the last years, similarities and interactions in acute transmission and sensitization processes between itch and pain were described [13]–[14]. In imaging studies of pain, women presented a higher activity in prefrontal, somatosensory and parietal gyri as well as in insula, dorsolateral prefrontal cortex (DLPFC), cingulate cortex, para−/hippocampus, cerebellum and thalamus even when the maximal pain intensity ratings were comparable between women and men [15]–[17].

Cognitive attention or distraction has a major influence on pain or itch perception. Distraction as a cognitive factor shifting the subject’s attention away from clinical pain or itch can reduce itch and pain intensity [18]–[21]. Keogh at al. [22] reported that men were more easily distractible than women from experienced pain. In another study examining the effect of smoking and distraction on pain sensitivity, men had less pain during distraction [23]. However, in a distraction study using local analgesia for oro-dental injections there was no sex difference in pain reduction [24].

To our knowledge, there is no paper published in the current literature on sex differences in central itch perception and impact of distraction on brain activity. Based on previous results in pain research and since it was discussed that pain and itch processing are comparable [14] we hypothesized that on the psychophysical level females will present higher itch intensities and desire to scratch that will decrease during distraction. In BOLD fMRI, females will show up-regulated activities of prefrontal, somatosensory and parietal gyri as well as in the insula, dorsolateral prefrontal cortex (DLPFC), cingulate cortex, para−/hippocampus, cerebellum and thalamus in response to itch when compared to men. Moreover, we assume that distraction will result in a more pronounced itch intensity reduction in men as compared to women.

## Methods

### Subjects

33 healthy subjects without history of chronic disease, allergy or mental illness were included in the study (17 women, mean ± SD age 24.3±2.8 years, 16 men, mean ± SD age 26.8±4.1 years). 28 participants were right handed, 5 left handed (3 women). All subjects provided written informed consent. The study was approved by the local Ethics Committee of the Medical Faculty of Muenster, Germany.

### Induction of Itch Sensation

Itch was induced by applying histamine intradermally via microdialysis fibers. Prior to fMRI, two microdialysis fibers (0.4 mm diameter, cut-off: 3.000 kDA, Asahi Plasmaflow) were placed intracutaneously in the left ventral forearm and two in the left medial lower leg using a 25-gauge canula. The fibers were filled with Ringer Solution (B. Braun, Melsungen, Germany) before intracutaneous placement. After placement, the skin was cooled by an ice bag for 5 minutes to reduce pain due to needle insertion. No local anesthesia was used. The distance between the fibers at each extremity was about 2.5 cm, the intradermal length was about 1 cm. After the subjects were placed in the scanner chamber, all fibers were controlled for correct placement, and skin temperature was checked to avoid a reduced itch sensation due to cold skin.

During the fMRI scanning, itch was induced by 0.5 ml of 10^−4^ M sterile histamine solution (Sigma, Deisenhofen, Germany) perfused by hand over 240 sec with a 2 ml syringe. The itch stimulation was terminated by perfusion of 0.7 ml 2% xylocaine-solution (Astra Zeneca, Wedel, Germany) and additional placement of an ice bag on the corresponding skin area for 90 sec.

During a second session the same experiment was repeated with 10 of the 33 volunteers (5 females, 5 males) of the previous experiment about 24 months after the first experiment. In this control condition histamine and xylocaine were replaced by physiological saline solution (0,9% NaCl solution) to serve as control for the ‘Stroop’ condition.

### Imaging and Experimental Protocol

Magnetic resonance imaging was performed with a 3T-scanner (Gyroscan, Philips, Best, The Netherlands) using a standard receiver head coil. For each subject, 844 echo-planar volumes (EPI) were obtained (TR = 2.5 sec, TE = 35 ms, flip angle 90°, matrix dimensions 64×64 field of view FOV = 210 mm and 36 oblique slices parallel to the AC-PC line. Slice thickness was 3.6 mm with a pixel size of 3.6×3.6×3.6 mm).

**Figure 1:**
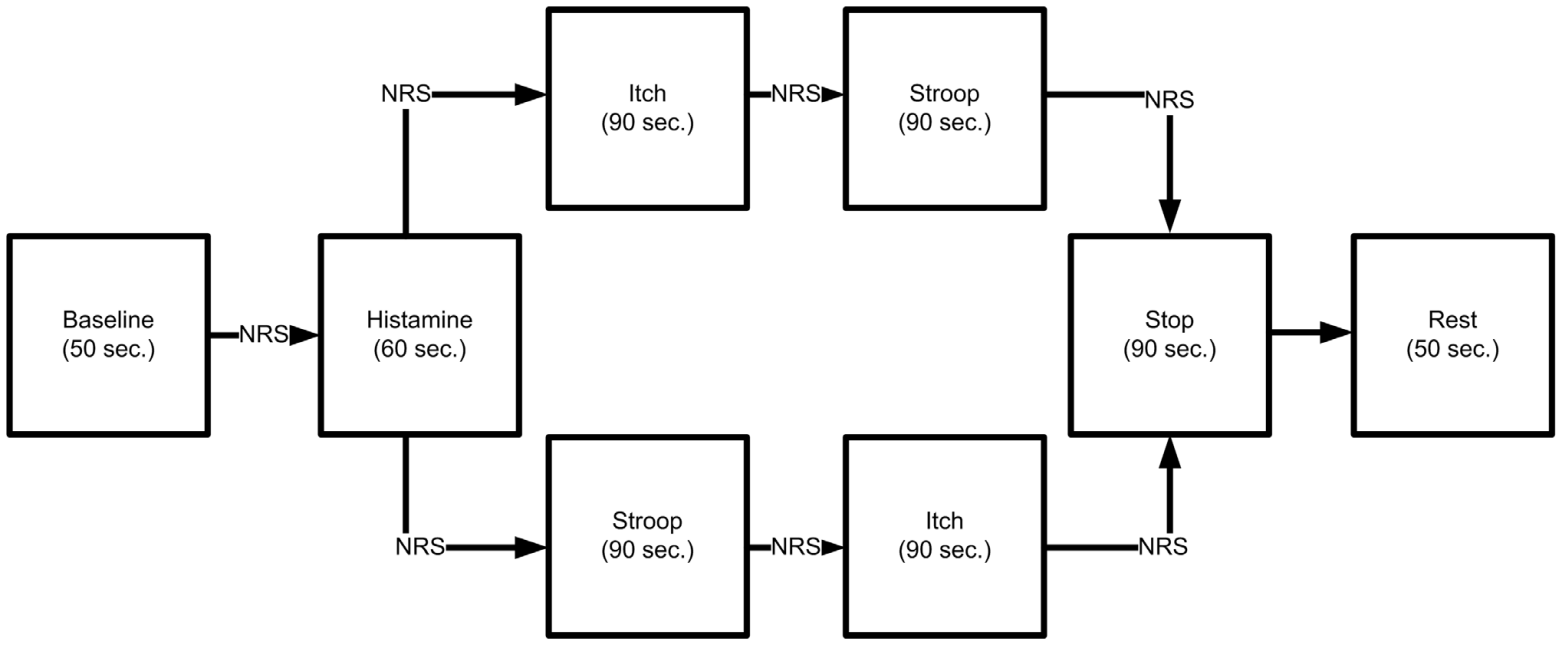
Experimental block design of itch stimulation. After a baseline period, histamine was injected on the forearm. After 60‘itch’ condition began, and the Stroop task had to be performed (90 s each) (A1_J+A1_S). Then, itch was terminated by xylocaine and an ice bag (90 s, upper panels). After a break, this procedure was carried out on the lower leg. To avoid habituation, the order of the second run was switched to first ‘Stroop’ and then the ‘itch’ condition (lower panels) (B1_S+B1_J). The procedure ‘Stroop-itch’ was carried out on the forearm (third run; A2_S+A2_J) and finally the procedure ‘itch-Stroop’ was carried out on the lower leg (fourth run; B2_J+B2_S). Thus, the tests were carried out in two different orders, once on the forearm and once on the lower leg. In a second experiment with 10 participants of the former study, histamine and xylocaine were replaced by saline solution. *NRS = numeric rating scale. Between each test block, the intensity of the itch sensation, the desire to scratch, and the experienced pain was determined with help of a numeric rating scale. **A1_J = first run forearm during ‘itch’, A1_S = first run forearm during ‘Stroop’, B1_J = first run lower leg during ‘itch’, B1_S = first run lower leg during ‘Stroop’, A2_J = second run forearm during ‘itch’, A2_S = second run forearm during ‘Stroop’, B2_J = second run lower leg during ‘itch’, B2_S = second run lower leg during ‘Stroop’.

During continuous fMRI scanning of the brain, the experimental itch stimulation was performed four times (see Figure 1): Histamine perfusion was initiated after a ‘baseline’ condition (50 seconds) of no sensory stimulation. 60 seconds later, when itch sensation had started, either a 90 seconds period of ‘itch’ condition or a 90 seconds period of ‘Stroop’ condition followed. During ‘baseline’ and ‘itch’ condition subjects were asked to look at a black cross projected on a screen via a mirror fixed on the head coil. During ‘Stroop’ condition, subjects had to perform a pseudorandomized colour Stroop task, deciding during 2.5 seconds whether the colour of the letters corresponds to the colour-word. Congruent tasks, e.g. “blue” was written in blue letters, were alternated with incongruent tasks, e.g. “red” was written in green. The subjects could give their decision, whether the colour of the letters corresponds to the word or not via a computer mouse. They moved the cursor to ‘yes’ for congruent tasks or ‘no’ for incongruent tasks. After one run composing of one ‘itch’ and one ‘Stroop’ condition, itch was terminated by xylocaine perfusion plus an ice pack on the site of stimulation during a period of 90 seconds. An additional resting period of 50 seconds followed to allow normalization of brain activation before another run started.

To avoid habituation effects, the order of both conditions (‘itch’ or ‘Stroop’ condition) was changed within and between the forearm and leg runs. This resulted in 4 runs in total, two times stimulation of the forearm (A1 =  first run started with the ‘itch’ condition, A2 =  the second run started with the ‘Stroop’ condition) and the lower leg (B1 =  first run started with the ‘Stroop’ condition, B2 =  second run started with the ‘itch’ condition).

### Psychophysical Measurements

After every experimental condition (‘baseline’, ‘itch’, ‘Stroop’) subjects were asked to rate their intensity of itch, desire to scratch and intensity of pain. Subjects presented their ratings via a numeric rating scale (NRS) ranging from 0 (no itch sensation/no desire to scratch/no pain sensation) to 10 (most intense itch/most intense desire to scratch/most intense pain imaginable) by using the computer mouse. In addition to these measurements, subjects were asked about their emotional valence (tenseness) and their arousal by a self-assessment manikin (SAM) [25] at the start and at the end of the experiment.

Statistics were performed by PASW 21.0 (SPSS Inc., Chicago, IL, USA). We used a general linear model of repeated measurements with the factors ‘condition’ (‘itch’, ‘Stroop’) and ‘localisation’ (two runs at the forearm and two runs at the lower leg) with ‘sex’ (female, male) as between-subject factor and post-hoc t-tests for independent variables.

We also calculated reaction times (mean ± SD) and error rates for congruent and non-congruent data colour word pairs separately. We used a univariate analysis of variance with ‘sex’ and ‘localisation’ as factors and reaction time as dependent variable and Bonferroni post-hoc test to analyse significant interactions of localisation with sex. Furthermore, we used t-tests for dependent variables to measure pre and post differences of valence and arousal. For the saline (NaCl) experiments we used Mann-Whitney-U-Tests to test in a pilot approach for sex-specific differences.

### Imaging Data Analysis

Functional images were analysed using the general linear model [26] for block designs in SPM8 (Welcome Department of Imaging Neuroscience; London, UK; www.fil.ion.ucl.ac.uk/spm). All images were realigned, normalized to an EPI-template (resulting voxel size of 2 mm), spatially smoothed (8 mm FWHM kernel), and high-pass filtered (128 s).

### First Level Analysis

For each subject data 2 conditions were defined: ‘itch’ condition (36 scans) and ‘Stroop’ condition (36 scans) with two runs at 2 localisations, resulting in 8 conditions in total. Realignment parameters were integrated as regressors into the model. For each subject the following contrasts were determined as a function of BOLD-signal changes of each single condition: ‘itch_A1’, ‘itch_A2’, ‘itch_B1’, ‘itch_B2’, ‘Stroop_A1’, ‘Stroop_A2’, ‘Stroop_B1’, ‘Stroop_B2’.

### Second Level Analysis

We used a SPM8 ‘full factorial’ design. The individual BOLD-contrasts were transferred into a 3-factorial ANOVA (factors *condition* (‘Stroop’, ‘itch‘, 2 levels, within subject factor), *sex* (‘female’, ‘male’, 2 levels, between subject factor) and *localisation* (‘forearm’ (A1+A2), ‘lower leg’ (B1+B2), 2 levels, within subject factor). According to our hypotheses the main effect of ‘sex’ and the interactions between ‘sex’ and the other factors were assessed. In addition, we performed differential post-hoc t-tests ‘female’<‘male’ and ‘female’>‘male’ for ‘itch’ and ‘Stroop’ condition and in relation to the different localisations separately. We used a Monte Carlo simulation to establish an appropriate voxel contiguity threshold [27]. Assuming an individual voxel type I error of p<0.005, a cluster extent of 70 contiguous resampled voxels or an individual voxel type I error of p<0.001, a cluster extent of 47 contiguous resampled voxels was indicated as sufficient to correct for multiple voxel comparisons at p<0.05 with our given scanner parameters. The saline experiments were analysed in the same manner.

Furthermore, we performed multiple regression analyses of each contrast (‘itch_A1’, ‘itch_A2’, ‘itch_B1’, ‘itch_B2’, ‘Stroop_A1’, ‘Stroop_A2’, ‘Stroop_B1’, ‘Stroop_B2’) for females and males separately to examine positive and negative correlations of the psychophysical data (itch intensity and desire to scratch) in relation to BOLD brain activities.

## Results

### Psychophysical and Neurobehavioral Data

There was no sex-specific difference between arousal and valence at the beginning and at the end of the experiment.

In our general linear model with the psychophysical data there was a significant main effect of ‘localisation’ (F = 8.0; p = 0.001) and ‘sex’ (F = 4.5, p = 0.042), but not for ‘condition’ (F = 0.6; p = n.s.) for itch intensity. Furthermore, we found a significant interaction of ‘localisation’×‘sex’ (F = 3.4; p = 0.031), ‘condition’×‘localisation’ (F = 9.9; p = <0.0001) and ‘condition’×‘localisation’×‘sex’ (F = 3.1; p = 0.041), but not for ‘condition’×‘sex’ (F = 0.022; p = n.s.).

Concerning the desire to scratch, there was also a significant effect of ‘localisation’ (F = 8.95; p = <0.0001) but not for ‘sex’ (F = 0.6; p = n.s.) or ‘condition’ (F = 0.5; p = n.s.). The interaction of ‘condition’×‘localisation’ was significant (F = 8.87; p = <0.0001), but not for ‘condition’×‘sex’ (F = 0.16; p = n.s.), ‘condition’×‘localisation’×‘sex’ (F = 1.6; p = n.s.) and ‘localisation’×‘sex’ (F = 2.5; p = n.s.). For pain intensities no significant main effects were found. There were no sex-specific differences over all variables during saline perfusion.

Generally, females depicted mean higher itch intensities compared to males during the ‘itch’ condition over the course of the experiment (females: mean itch intensities: 4.3±1.8 and males: 3.1±1.3, students t-test: T = 2.1, p = 0.045). There was no significant sex difference in the mean desire to scratch over the total course of the experiment.

**Figure 2:**
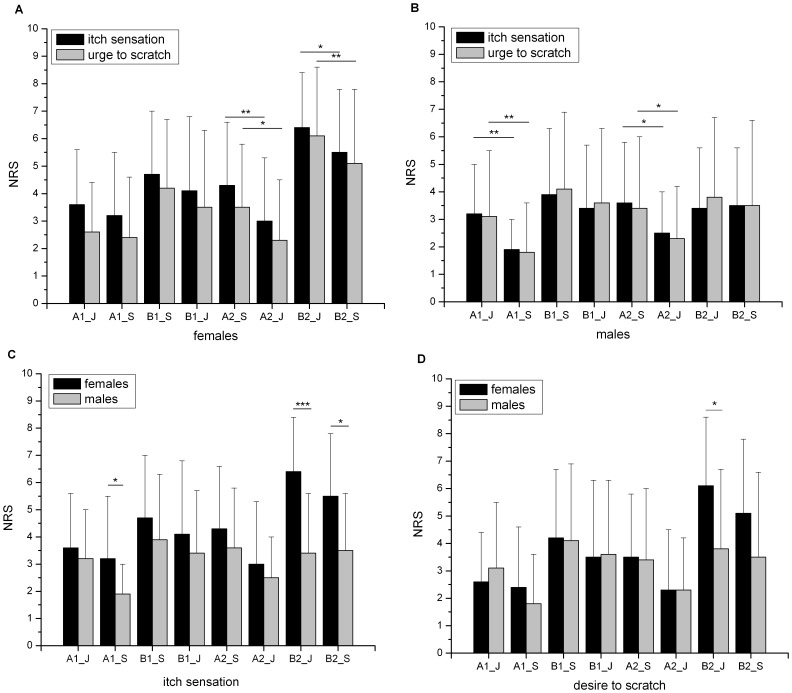
Sex-specific differences in itch intensity and desire to scratch. A) Plot of the female’s itch intensity and desire to scratch during ‘itch’ and ‘Stroop’ condition and B) Plot of male’s itch intensity and desire to scratch during ‘itch’ and ‘Stroop’ condition. In C) sex-specific comparison of itch intensity during ‘itch’ and ‘Stroop’ condition und in D) sex-specific comparison of desire to scratch during ‘itch’ and ‘Stroop’ condition are plotted. ** = p≤0.05, ** = p≤0.01, *** = p≤0.0001. **A1_J = first run forearm during ‘itch’, A1_S = first run forearm during ‘Stroop’, B1_J = first run lower leg during ‘itch’, B1_S = first run lower leg during ‘Stroop’, A2_J = second run forearm during ‘itch’, A2_S = second run forearm during ‘Stroop’, B2_J = second run lower leg during ‘itch’, B2_S = second run lower leg during ‘Stroop’.

A more detailed analyses of the significant interaction ‘condition’×‘localisation’×‘sex’ indicated that females had higher itch intensities and desire to scratch during ‘itch’ compared to the ‘Stroop’ condition during stimulation at the lower leg when ‘itch’ was followed by the ‘Stroop’ condition (B2, p = 0.039 for itch sensation, p = 0.01 for desire to scratch). Surprisingly, during the two runs, where the ‘Stroop’ condition was first, a subsequent reduction of the itch sensation and desire to scratch during ‘itch’ condition was observed (see A2 in Figure 2A). Parallel, females also depicted lower pain during distraction by ‘Stroop’ when compared to ‘itch’ intensities in B2 (‘itch’: 2.4; ‘Stroop’: 1.8; p = 0.046), in contrast, pain intensities during ‘Stroop’ were higher when compared to ‘itch’ when ‘itch’ followed the ‘Stroop’ condition in A2 (‘Stroop’: 1.8; ‘itch’: 1.2; p = 0.044).

In contrast to females, males presented a different pattern of itch sensation. Here, we observed significant differences between ‘itch’ and ‘Stroop’ only at the forearms. In both runs, the second part of stimulation (‘itch’ or ‘Stroop’) lead to a reduction of itch sensation and desire to scratch (see Figure 2B). There were no significant differences between ‘itch’ and ‘Stroop’ for pain intensities.

To summarize, a strong location specific effect of sex was observed. Females exhibited higher itch intensities and a stronger desire to scratch during lower leg stimulation (B2) during ‘itch’ and ‘Stroop’ condition. In contrast, men showed a stronger reduction of itch during distraction for the first run in the forearms (A1). For further details see Table 1 and Figures 2C and D.

**Table 1:** Students two-sided t-test of psychophysical data for females and males (NRS) during histamine perfusion.

	Females				Males			t value	p
*Itch sensation*									
	Mean			SD	Mean		SD		
A1_J*	3.6			2.0	3.2		1.8	0.6	n.s.
A1_S*	3.2			2.3	1.9		1.1	2.08	0.049
B1_S*	4.7			2.3	3.9		2.4	1.02	n.s.
B1_J*	4.1			2.7	3.4		2.3	0.77	n.s.
A2_S*	4.3			2.3	3.6		2.2	0.84	n.s.
A2_J*	3.0			2.3	2.5		1.5	0.72	n.s.
B2_J*	6.4			1.9	3.4		2.2	4.3	<0.0001
B2_S*	5.5			2.3	3.5		2.1	2.6	0.013
*Desire to scratch*									
A1_J*	2.6		1.8		3.1	2.4		−0.56	n.s.
A1_S*	2.4		2.2		1.8	1.8		0.85	n.s.
B1_S*	4.2		2.5		4.1	2.8		0.12	n.s.
B1_J*	3.5		2.8		3.6	2.7		−0.03	n.s.
A2_S*	3.5		2.3		3.4	2.6		0.18	n.s.
A2_J*	2.3		2.2		2.3	1.9		0.06	n.s.
B2_J*	6.1		2.5		3.8	2.9		2.47	0.019
B2_S*	5.1		2.7		3.5	3.1		1.54	n.s.
Pain sensation									
A1_J*	1.5	2.2			1.4	1.8		0.22	n.s.
A1_S*	1.7	2.4			0.9	1.1		1.2	n.s.
B1_S*	2.1	2.2			1.9	1.7		0.27	n.s.
B1_J*	1.8	2.3			1.8	1.9		0.02	n.s.
A2_S*	1.8	2.3			2.1	2.3		−0.3	n.s.
A2_J*	1.2	1.8			1.9	3.0		−0.82	n.s.
B2_J*	2.4	2.4			1.5	1.5		1.3	n.s.
B2_S*	1.8	2.1			1.8	2.3		0.1	n.s.

* A1_J = first run forearm during ‘itch’, A1_S = first run forearm during ‘Stroop’, B1_J = first run lower leg during ‘itch’, B1_ S = first run lower leg during ‘Stroop’, A2_J = second run forearm during ‘itch’, A2_ S = second run forearm during ‘Stroop’, B2_J = second run lower leg during ‘itch’, B2_ S = second run lower leg during ‘Stroop’.

During the control experiments there were only very low intensities of itch, pain and desire to scratch with no sex-specific differences. For further details see Table S1.

#### Influence of distraction - reaction time and error rates

Every participant performed a ‘Stroop’ task with 36 trials during each run, so 4 ‘Stroop’ tasks in total. 4752 trials were performed (144×33 participants = 4752). We deleted all data with reaction times <300 msec and>3000 msec where participants pushed the mouse button too late or too early (n = 170). In total we included 4582 trials, 2364 for females (reaction time: 912.05±327.8 msec) and 2218 for males (923.4±315.8 msec; p = n.s.) There were 4158 correct answers (90.7%), 2145 for females (90.7%, reaction time: 909.1±320.1 msec) and 2013 for males (90.8%, reaction time: 920.0±311.8 msec). For further analyses the data of correct answers only were used. The low drop-out rate of around 3% and an error rate of around 10% indicate that subjects performed the task as expected and were distracted.

In an univariate analysis of variance with ‘sex’ and ‘localisation’ as factors and reaction time as dependant variable and Bonferroni post-hoc tests there were significant differences for ‘localisation’ (p≤0.0001) but not for ‘sex’ (p = n.s.) nor for their interactions (p = n.s.). The reaction time decreased over the course of the experiment due to a practice effect (Table 2).

**Table 2:** Mean reaction time of correct answers (mean ± SD) in the Stroop test by univariate variance analysis and post-hoc Bonferroni correction for all participants.

Localisation	Reaction time (msec) -all participants-	Bonferroni correction**	Reaction time (msec)-females-	Reaction time (msec)-males-
**A1** *	997.0±338.5 (n = 993)	A1> B1***	1003.7±356.0 (n = 508)	989.9±319.1 (n = 485)
		A1> A2***		
		A1> B2***		
**B1** *	906.4±297.8 (n = 1042)	B1 = A2	892.2±280.9 (n = 545)	922.1±314.9 (n = 497)
		B1> B2**		
**A2** *	903.2±318.2 (n = 1040)	A2> B2**	896.3±325.5 (n = 541)	910.5±310.2 (n = 499)
**B2** *	854.9±294.9 (n = 1083)		848.4±298.9 (n = 551)	861.8±290.7 (n = 532)

Reduction of reaction times can be clearly depicted over the course of the experiment due to a practice effect. Reaction times did not differ between males and females.

* A1 =  first run forearm; A2 =  second run forearm; B1 =  first run lower leg; B2 =  second run lower leg.

** = p≤0.01;

*** = p≤0.0001.

### FMRI Data

#### BOLD cluster analysis

Sex-specific differences in brain activities were postulated. These were tested by a 3-factorial ANOVA with the between subject factor ‘sex’ and the dependent within subject factors ‘condition’ and ‘localisation’. A significant main effect of ‘sex’ was found (Table S2) in mostly frontal brain areas, the anterior cingulate cortex, the lentiform nucleus and the cerebellum.

This main effect can be explained by mainly a higher brain activity of women when compared to men (females>males) during ‘itch’ (Table 3) and during ‘Stroop’ (Table 4).

**Table 3:** Differential contrasts (t-tests) of sex-specific activation under ‘itch’ condition (uncorrected, p<0.001, with a voxel threshold k>47).

Region	k	Z-score	p (uncorr.)	coordinates (x y z mm)		
**females>males**						
Left inf. parietal lobule (BA 40)*	267	4.49	<0.0001	−46	−56	52
Left inf. frontal gyrus (BA 47)*	556	4.41	<0.0001	−46	22	−14
		3.91	<0.0001	−36	28	−6
Right inf. frontal gyrus (BA 47)*	547	4.36	<0.0001	44	26	−4
		4.16	<0.0001	56	18	−2
Right inf. frontal gyrus (BA 45)*		3.83	<0.0001	36	24	4
Right sup. frontal gyrus; SMA (BA 6)*	91	4.26	<0.0001	4	20	62
Right inf. parietal lobule (BA 40)*	198	4.25	<0.0001	48	−56	54
		3.6	<0.0001	56	−46	48
Right middle occipital gyrus (BA 18)	86	4.16	<0.0001	32	−80	4
Right medial frontal gyrus (BA 6)	531	4.03	<0.0001	8	26	38
Right medial frontal gyrus (BA 9)		3.82	<0.0001	2	36	32
Left medial frontal gyrus (BA 6)		3.77	<0.0001	−4	28	36
Left cerebellum	158	3.98	<0.0001	−6	−52	−32
Right cerebellum		3.84	<0.0001	2	−54	−32
Right caudate body	72	3.93	<0.0001	20	20	8
Right middle frontal gyrus (BA 8)	170	3.85	<0.0001	38	38	38
Right middle frontal gyrus; DLPFC (BA 9)*		3.8	<0.0001	36	26	36
Left lentiform nucleus	106	3.63	<0.0001	−20	8	12
		3.35	<0.0001	−16	12	4
		3.14	0.001	−20	20	4
Left middle frontal gyrus (BA 6)	75	3.62	<0.0001	−42	6	58
		3.55	<0.0001	−38	0	62
**males>females**						
Left cuneus (BA 18)	143	4.45	<0.0001	−12	−102	4
Left cuneus (BA 19)		3.45	<0.0001	0	−88	26
		3.24	0.001	−10	−96	30

* inf. = inferior, sup. = superior, DLPFC = dorsolateral prefrontal cortex, SMA = supplementary motor area.

**Table 4:** Differential contrasts (t-tests) of sex-specific activation under ‘Stroop’ condition (uncorrected, p<0.001, with a voxel threshold k>47).

Region	k	Z-score	p (uncorr.)	coordinates (x y z mm)		
**females>males**						
Left medial frontal gyrus (BA 6)	62	3.63	<0.0001	−4	34	34
Right middle frontal gyrus (BA 8)	71	3.6	<0.0001	34	40	42
Right middle frontal gyrus; DLPFC (BA 9)*		3.6	<0.0001	46	36	36
Left inf. frontal gyrus (BA 47)*	75	3.55	<0.0001	−38	16	−12
Left sup. temporal gyrus (BA 38)*		3.44	<0.0001	−34	22	−26
**males>females**						
Right cerebellum	51	3.85	<0.0001	28	−42	−26

* inf. = inferior, sup. = superior, DLPFC = dorsolateral prefrontal cortex.

During ‘itch’ mostly frontal brain areas, including the SMA and the DLPFC as well as the cerebellum and the lentiform nucleus were more active in females than in males. In contrast, males presented only a higher activation of the left cuneus when compared to women. Furthermore, significant interactions of ‘sex’×‘localisation’ and of ‘sex’×‘condition’ were observed (Tables S3–S4).

While the interaction of ‘sex’×‘condition’ was related to an activation of temporal and occipital gyri (BA 18, 21, 38), the cuneus (BA 19), the post, cingulate gyrus (BA 30) and the right cerebellum, the interaction of ‘sex’×‘localisation’ revealed an activation of the right precentral gyrus (BA 6), the right thalamus, the lingual gyrus and the right insula. The interaction of all three factors did not show any threshold clusters.

During NaCl perfusion no main effect of ‘sex’ was seen and therefore no threshold clusters detectable.

As there were sex-specific differences in the psychophysical data for different conditions and different localisation of stimulation, we also assessed the influence of the stimulation site on brain activity. Interestingly, during stimulations at the forearm (A1+ A2), there were only little differences between males and females during ‘itch’. Here, males depicted a higher activation of occipital gyrus and cuneus (BA 18, 19) in contrast to females. The latter had higher activations in frontal brain areas (BA 47) and the cerebellum (Table 5). During ‘Stroop’ there were no sex-specific differences detectable.

**Table 5:** Differential contrasts (t-test) of sex-specific activation during forearm stimulation during ‘itch’ condition (uncorrected, p<0.001, with a voxel threshold k>47).

Region	k	Z-score	p (uncorr.)	coordinates(x y z mm)		
**females>males**						
Left cerebellum	75	4.15	<0.0001	−6	−52	−36
Left inf. frontal gyrus (BA 47)*	134	4.01	<0.0001	−48	22	−14
**males>females**						
Left cuneus (BA 18)	285	4.32	<0.0001	−12	−100	4
Left middle occipital gyrus (BA 18)		3.75	<0.0001	−10	−90	12
Left cuneus (BA 19)		3.66	<0.0001	0	−88	24

* inf. = inferior.

**Figure 3:**
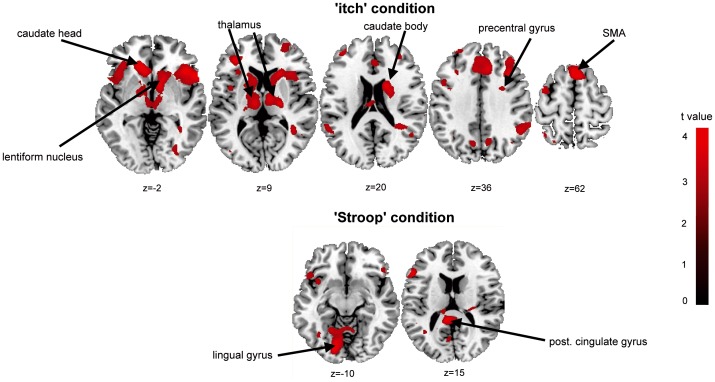
Sex-specific differences in fMRI BOLD response during ‘itch’ and ‘Stroop’ condition during stimulation at the lower legs. The figure shows the different brain activity maps during ‘itch’ and ‘Stroop’ condition for ‘females’>‘males’. The results are corrected for multiple comparisons (uncorrected, p<0.001, voxel threshold k>47 voxels). *SMA = supplementary motor area; post. = posterior.

Stimulations at the lower leg and in line with the psychophysical data (Table 1, Figure 2C) resulted in higher brain activity in females in contrast to males (females>males) but not vice versa during ‘itch’ condition. Females presented higher activations of mostly frontal gyri, including SMA, parietal lobule (BA 40), the lentiform nucleus as well as the thalamus and precentral gyrus (BA 9) (Table 6 and Figure 3). The differences between both sexes in ‘Stroop’ condition were limited to a stronger activation in females in contrast to men in the left lingual gyrus, the left culmen and the left post. cingulate gyrus (BA 29) (Table 7 and Figure 3). The reverse contrast (males>females) did not show any brain activity.

**Table 6:** Differential contrasts (t-test) of sex-specific activation during lower leg stimulation during ‘itch’ condition (uncorrected, p<0.001, with a voxel threshold k>47).

Region	k	Z-score		p (uncorr.)	coordinates (x y z mm)			
**females>males**								
Left medial frontal gyrus (BA 10)	527	4.51	<0.0001		−16	32	−4	
Left inf. frontal gyrus (BA 47)*		3.93	<0.0001		−34	22	−14	
Left caudate head		3.77	<0.0001		−8	24	−2	
Left inf. parietal lobule (BA 40)*	226	4.46	<0.0001		−50	−54	54	
Left inf. parietal lobule (BA 7)*		3.35	<0.0001		−38	−62	42	
Right sup. frontal gyrus; SMA (BA 6)*	154	4.45	<0.0001		4	20	62	
Right inf. parietal lobule (BA 40)*	417	4.42	<0.0001		50	−54	54	
		3.88	<0.0001		60	−44	42	
Right supramarginal gyrus (BA 40)		3.42	<0.0001		50	−50	32	
Right lingual gyrus (BA 19)	63	4.17	<0.0001		30	−76	2	
Right inf. frontal gyrus (BA 47)*	405	4.11	<0.0001		56	18	−2	
		4.02	<0.0001		44	24	−4	
Right inf. frontal gyrus (BA 13)*		3.39	<0.0001		36	22	6	
Left middle frontal gyrus (BA 10)	58	3.98	<0.0001		−34	38	10	
Right medial frontal gyrus (BA 8)	231	3.75	<0.0001		10	30	42	
Right medial frontal gyrus (BA 9)		3.34	<0.0001		6	32	30	
Right medial frontal gyrus (BA 8)		3.29	<0.0001		4	38	42	
Right caudate body	86	3.71	<0.0001		18	4	20	
Right lentiform nucleus	117	3.65	<0.0001		10	6	−2	
		3.47	<0.0001		16	14	−4	
Right claustrum		3.18	<0.0001		22	22	−6	
Right thalamus	100	3.62	0.001		8	−10	4	
Right precentral gyrus (BA 9)	59	3.56	<0.0001		36	22	36	
Left thalamus	96	3.53	<0.0001		−10	−16	10	

* inf. = inferior, sup. = superior, SMA = supplementary motor area.

**Table 7:** Differential contrasts (t-test) of sex-specific activation during lower leg stimulation during ‘Stroop’ condition (uncorrected, p<0.001, with a voxel threshold k>47).

Region	k	Z-score	p (uncorr.)	coordinates (x y z mm)			
**females>males**							
Left lingual gyrus (BA 18)	185	4.04	<0.0001	−16	−82	−10	
Left culmen		3.42	<0.0001	−10	−68	−6	
Left posterior cingulate gyrus (BA 29)	67	3.94	<0.0001	−8	−42	16	
Right posterior cingulate gyrus (BA 29)		3.31	<0.0001	2	−40	14	

#### Regression analysis

**Figure 4:**
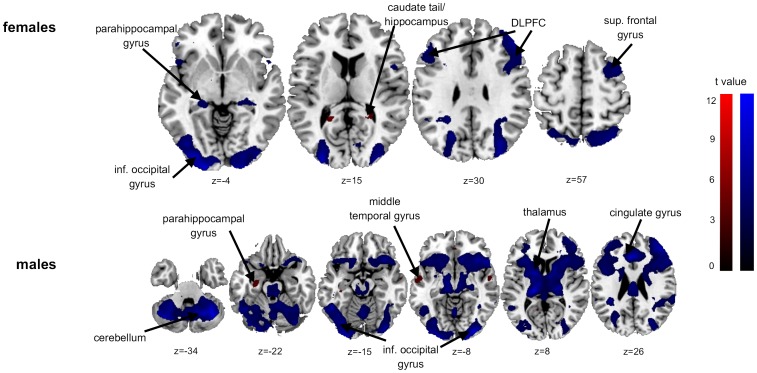
Correlation analysis of itch intensity ratings with brain activities during ‘itch’ condition (B2). The figure shows positive (in red) and negative (in blue) correlations of brain BOLD-activity with itch intensity ratings during second stimulation at the lower legs (B2) for females and males separately (Females: positive correlation: uncorrected, p<0.005, with a voxel threshold k>70, negative correlation: uncorrected, p<0.001, with a voxel threshold k>47; males: positive correlation: uncorrected, p<0.005, with a voxel threshold k>70, negative correlation: FWE corrected, with a voxel threshold k>10). *DLPFC = dorsolateral prefrontal cortex; inf. = inferior; sup. = superior.

Correlations analyses revealed comparable positive and negative correlations in the psychophysical data (itch intensity and desire to scratch) during ‘itch’ and ‘Stroop’ in A1, A2 and B1 (see Tables S5A – S5F for A1, A2 and B1, respectively). In line with psychophysical and BOLD brain activity (Tables 6 and 7, Figures 2 and 3) there were distinct sex-specific correlation differences during ‘itch’ and ‘Stroop’ during the second lower leg stimulation (B2). Females presented positive correlations of itch ratings during ‘itch’ condition with activation intensities of the right hippocampus and the right caudate tail. There were negative correlations with activation intensities of occipital gyri (BA 18), frontal gyri (BA 6, 8, 9) including the DLPFC, the parahippocampal gyrus (BA 27), the inf. parietal lobule (BA 40) and the right cuneus (BA 17). Males presented positive correlations with the parahippocampal gyrus (BA 35) and the middle temporal gyrus (BA 21) and negative correlations with occipital gyri (BA 17, 18), the thalamus, the cerebellum, frontal gyri (BA 6, 8, 46) and the cingulate gyrus (BA 32) (Table 8, Figure 4).

**Table 8:** Sex-specific correlations of itch intensity with brain activity during second lower leg stimulation (B2) during ‘itch’ condition.

Region	k	Z-score	p	coordinates (x y z mm)		
**females**						
*positive correlation* **						
Right hippocampus	122	3.58	<0.0001	24	−44	10
Right caudate tail		3.37	<0.0001	16	−36	20
Negative correlation*						
Left middle occipital gyrus (BA 18)	12893	5.90	<0.0001	−28	−98	6
Left inferior occipital gyrus (BA 18)		5.56	<0.0001	−30	−90	−6
Right middle frontal gyrus; DLPFC (BA 9)†	857	4.87	<0.0001	46	36	36
		4.37	<0.0001	54	28	30
Right sup. frontal gyrus; DLPFC (BA 9)†		4.01	<0.0001	44	44	32
Right sup. frontal gyrus (BA 6)†	555	4.09	<0.0001	28	8	66
Right middle frontal gyrus (BA 6)		3.92	<0.0001	28	4	50
		3.91	<0.0001	38	6	56
Left inf. parietal lobule (BA 40)†	99	3.66	<0.0001	−54	−48	38
Left middle frontal gyrus; DLPFC (BA 9)†	294	3.61	<0.0001	−48	30	30
		3.43	<0.0001	−48	18	32
Left middle frontal gyrus (BA 8)		3.27	0.001	−54	14	40
Left parahippocampal gyrus (BA 27)	49	3.55	<0.0001	−22	−30	−4
Right cuneus (BA 17)	57	3.54	<0.0001	14	−78	8
Left medial frontal gyrus (BA 8)	63	3.27	0.001	0	20	44
**males**						
*positive correlation* **						
Left parahippocampal gyrus (BA 35)	86	3.27	0.001	−26	−14	−22
Left middle temporal gyrus (BA 21)	94	3.01	0.001	−58	−12	−8
		2.8	0.003	−54	0	−10
*negative correlation* ***						
Left thalamus	49	5.77	<0.0001	−18	−26	8
		5.26	<0.0001	−22	−30	2
Right cerebellum	112	5.66	<0.0001	38	−60	−32
		4.99	<0.0001	38	−72	−30
Left inf. occipital gyrus (BA 17)†	32	5.47	<0.0001	−26	−98	−10
Left inf. occipital gyrus (BA 18)†		4.98	<0.0001	−32	−94	−16
Right middle frontal gyrus; DLPFC (BA 46)†	65	5.42	<0.0001	44	48	22
Right inf. occipital gyrus (BA 18)†	44	5.36	<0.0001	32	−92	−6
Right lingual gyrus (BA 17)	20	5.26	<0.0001	20	−100	−10
Right medial frontal gyrus (BA 8)	14	5.19	<0.0001	8	20	44
Right inf. parietal lobule (BA 40)†	19	5.14	<0.0001	44	−46	40
Right middle frontal gyrus (BA 6)	25	5.12	<0.0001	32	0	50
Left cingulate gyrus (BA 32)	12	5.11	<0.0001	−4	28	26
Right cerebellum	11	5.10	<0.0001	26	−62	−34

(*uncorrected, p<0.001, with a voxel threshold k>47;

** uncorrected, p<0.005, with a voxel threshold k>70;

*** FWE corrected, p<0.05, with a voxel threshold k>10).

† inf. = inferior, sup. = superior, DLPFC = dorsolateral prefrontal cortex.

**Figure 5:**
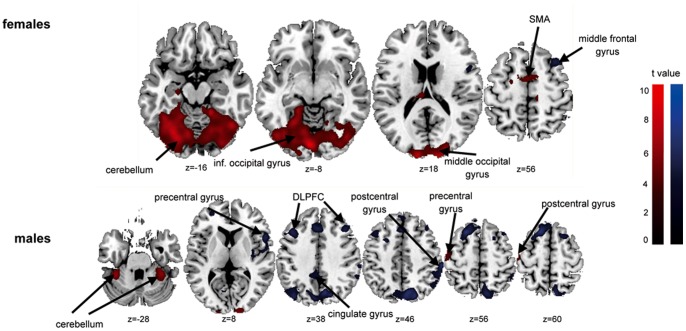
Correlation analysis of itch intensity ratings with brain activities during ‘Stroop’ condition (B2). The figure shows positive (in red) and negative (in blue) correlations of brain BOLD-activity with itch intensity ratings during second stimulation at the lower legs (B2), for females and males separately. The results are corrected for multiple comparisons (uncorrected, p<0.001, voxel threshold k>47 voxels). *SMA = supplementary motor area; DLPFC = dorsolateral prefrontal cortex; inf. = inferior.

Females showed positive correlations of itch ratings during ‘Stroop’ with activation intensities of the occipital gyrus (BA 18), cerebellum and medial frontal gyrus (SMA) and negative correlations with parietal (BA 40) and frontal gyri (BA 6). Males had positive correlations not only with occipital gyri (BA 18) and cerebellum but also with precentral and postcentral gyri. Furthermore there were extended negative correlations with frontal gyri (including DLPFC, BA 6, 8, 9, 46, 47), temporal (BA 22, 39) and parietal gyri (BA 40) as well as precentral (BA 44) and postcentral gyri (BA 2) (Table 9, Figure 5).

**Table 9:** Sex-specific correlations of itch intensity with brain activity during second lower leg stimulation (B2) during ‘Stroop’ condition (uncorrected, p<0.001, with a voxel threshold k>47).

Region	k	Z-score	p (uncorr.)	coordinates (x y z mm)				
**females**								
*positive correlation*								
Left lingual gyrus (BA 18)	8224	5.38	<0.0001	−4		−84		−8
Left inferior occipital gyrus (BA 18)		5.22	<0.0001	−36		−84		−2
Left cerebellum		5.04	<0.0001	−28		−70		−16
Left middle occipital gyrus (BA 18)	1025	5.11	<0.0001	−12		−98		14
Right middle occipital gyrus (BA 18)		4.65	<0.0001	14		−100		18
		4.26	<0.0001	24		−90		18
Left hypothalamus	72	3.76	<0.0001	−2		−4		−4
Right medial frontal gyrus; SMA (BA 6)*		3.45	<0.0001	6		0		56
*negative correlation*								
Right inf. parietal lobule (BA 40)	222	4.44	<0.0001	48		−50		48
Right middle frontal gyrus (BA 6)	98	3.87	<0.0001	34		18		58
**males**								
*positive correlation*								
Right fusiform gyrus (BA 19)	532	4.77	<0.0001	42			−66	−6
Right middle occipital gyrus (BA 18)		4.32	<0.0001	32			−80	−6
Right cerebellum		3.61	<0.0001	28			−78	−14
Right cuneus (BA 18)	110	4.23	<0.0001	16			−100	12
Left cerebellum	150	4.10	<0.0001	−36			−44	−26
Right cerebellum	161	4.07	<0.0001	36			−44	−28
Left middle occipital gyrus (BA 18)	71	3.75	<0.0001	−40			−80	−8
Left cuneus (BA 18)	53	3.58	<0.0001	−18			−100	12
Left precentral gyrus (BA 6)	58	3.52	<0.0001	−50			−6	56
Left postcentral gyrus (BA 3)		3.50	<0.0001	−50			−18	60
*negative correlation*								
Left superior frontal gyrus (BA 6)	487	4.99	<0.0001	−14		22		62
Left middle frontal gyrus (BA 6)		4.71	<0.0001	−20		22		56
Left superior frontal gyrus (BA 6)		3.85	<0.0001	−24		10		48
Left middle temporal gyrus (BA 39)	345	4.70	<0.0001	−36		−70		32
Left angular gyrus (BA 39)		3.17	0.001	−52		−70		38
Right precuneus (BA 7)	1236	4.65	<0.0001	14		−72		50
		4.59	<0.0001	8		−74		42
		4.47	<0.0001	20		−74		36
Right inferior frontal gyrus (BA 47)	502	4.57	<0.0001	50		28		−16
Right precentral gyrus (BA 44)		3.89	<0.0001	56		14		8
Right superior temporal gyrus (BA 22)		3.70	<0.0001	62		10		2
Left inferior frontal gyrus(BA 47)	915	4.44	<0.0001	−50		24		−10
Left middle frontal gyrus (BA 47)		4.28	<0.0001	−44		34		−6
Left medial frontal gyrus (BA 9)	600	4.43	<0.0001	−4		32		32
Left medial frontal gyrus (BA 8)		3.88	<0.0001	0		26		40
Right postcentral gyrus (BA 2)	349	4.14	<0.0001	60		−28		46
Right inferior parietal lobule (BA 40)		3.88	<0.0001	56		−44		50
		3.36	<0.0001	46		−60		46
Left middle frontal gyrus (BA 8)	202	4.02	<0.0001	−40		22		40
Left middle frontal gyrus; DLPFC (BA 9)*		3.35	<0.0001	−52	20			38
Right middle frontal gyrus; DLPFC (BA 46)*	285	3.90	<0.0001	44	40			26
Right middle frontal gyrus; DLPFC (BA 9)*		3.84	<0.0001	42	28			36
Right superior frontal gyrus (BA 6)	50	3.65	<0.0001	16	16			62
Left cuneus (BA 18)	86	3.61	<0.0001	−10	−72			16
		3.25	0.001	0	−70			20
Left inferior frontal gyrus (BA 45)	79	3.60	<0.0001	−54	24			18
Left middle frontal gyrus (BA 10)	76	3.47	<0.0001	−28	50			10
Left superior frontal gyrus (BA 6)		3.21	0.001	−32	56			16
Left middle temporal gyrus (BA 39)	58	3.33	<0.0001	−52	−62			26
Left superior temporal gyrus (BA 39)		3.18	0.001	−60	−58			28
Left cingulate gyrus (BA 31)	59	3.31	<0.0001	−6	−44			38
Right cingulate gyrus (BA 31)		3.18	0.001	2	−48			40

* DLPFC = dorsolateral prefrontal cortex, SMA = supplementary motor area.

Results were comparable for the correlations with the desire to scratch ratings during B2.

## Discussion

### Psychophysical Data

In the present study, differences between females and males in itch intensity and desire to scratch in psychophysical data but also in central itch perception were found. In line with our first hypothesis, women generally presented higher itch intensities compared to men during ‘itch’ condition over the course of experiment. A more specific analysis of the interaction ‘sex’×‘condition’×‘localisation’ revealed higher itch intensities, desire to scratch and itch associated pain in women during experimental induced itch that can be reduced by distraction at the lower legs when ‘itch’ is followed by ‘Stroop’ (Figures 2C+D). In contrast, men depicted significant reduction of ‘itch’ by ‘Stroop’ at the forearms (Figure 2B). No sex differences were seen in the saline control condition.

The knowledge about sex-specific differences in itch perception is very limited at present. Ständer et al. [10] could show in a large sample of 1037 patients with chronic pruritus that females exhibited higher itch intensities on a visual analog scale and suffered more from itch than males. These results are well in line with the results of the present study. Similarly, larger histamine-induced wheal responses were found in females upon iontophoresis [28] compared to males. However, sex-specific itch ratings were not presented.

Remarkably, women also reported higher itch associated pain scores, too. This corroborates findings of our recent study, where women compared to men reported more often on localised itching occurring in attacks, with stinging, warmth, and painful qualities [10]. These findings also correspond to pain research. Here, it is already well known, that females appear to have higher pain sensitivities [29]–[30]. Similarly, it seems feasible to assume that the same may hold true for itch perception with females exhibiting a lower itch threshold and associated a lower threshold of desire to scratch compared to men.

Sex-specific differences were pronounced for the lower legs (Table 1, Figures 2C and D). Truini et al. [31] and Magerl et al. [28] could show previously that itch sensation increased from head to the lower extremities, pointing to region specific differences in itch perception. However they did not analyse itch ratings sex-specifically. The only study who investigated sex differences was performed by Bergeret et al. [32]. They only stimulated at the arms and not at the legs and did not find any sex-specific difference during itch stimulation. Our study corroborated their findings, since we also did not find sex-specific differences in itch perception during itch without distraction at the forearms.

Our finding of localised sex-specific differences may be explained by different itch receptor distribution between the sexes as postulated by Truini et al. [31]. This is also supported by our data in patients with chronic pruritus where itching could be reduced significantly more often in women by cold treatment and in men by heat treatment, indicating differences in receptors in the skin [10]. To date, it is still unclear however if the different clinical distribution of affected body areas is due to the underlying disease or if it might be due to sex differences in the anatomy of the skin.

Another sex-related difference was that itch sensation and desire to scratch could only be reduced by distraction at the lower legs in women and at the forearms in men in the two runs, where ‘itch’ was followed by ‘Stroop’. Somewhat unexpected was the observation that during the two runs, in which we first presented the distraction paradigm followed by itch sensation without distraction, a higher itch intensity was observed during ‘Stroop’ compared to ‘itch’ in the forearms (Figures 2A–D). This finding may be explained as follows: the effect of the distraction paradigm might last longer as previously assumed, so the itch sensation cannot develop sufficiently. As our distraction and itch sensation paradigm is quite short we cannot resolve how long this effect may last.

Our data did not indicate as previously hypothesized that itch intensities can be reduced more efficiently in men as compared to women by distraction. Depending on the localisation of the experimental induced itch women and men were distractible similarly. This is in contrast to pain results. For example, male adolescents used distraction as a coping strategy for chronic back pain [22]. This indicates that the different quality of itch sensation seems to provoke different sex-specific modulation systems than pain does.

During the control experiment with saline perfusion, there were no significant sex-specific differences, supporting the notion that our observed sex differences during itch and reduction by distraction are due to different perception of itch between the sexes.

### Central Itch Processing during Itch Sensation

Our hypothesis of sex-specific differences in brain activity was confirmed. Beside a significant main effect of ‘sex’ (Table S2), significant interactions of ‘sex’×‘localisation’ and of ‘sex’×‘condition’ were observed (Tables S3 and S4). The main effect of ‘sex’ can be explained by mainly a higher brain activity of women when compared to men (females>males) during ‘itch’ (Table 3) and during ‘Stroop’ (Table 4) in mostly frontal brain areas. The control experiments with saline perfusion did not show any main effect of ‘sex’, there were no activated clusters.

Since as to our knowledge this is the first study examining sex differences in central itch perception we cannot compare our results to other studies dealing with sex differences in itch, therefore our results were compared to pain results. Here, sex differences in frontal areas like the DLPFC were also reported. The DLPFC is well known to play a putative role of pain anticipation and is considered as “keeping pain out of mind” [33]. Benson et al. [34] found a higher activation of DLPFC in women during anticipation of pain. So in line with these findings it might be possible that women do not only have an enhanced recruitment of pain control mechanisms but also of itch control. It may also reflect observations that women suffer more from itch as reported in our recently published study on 1037 patients [10].

The higher experienced itch intensity and desire to scratch in women is also reflected in a higher activation of the cerebellum and supplementary motor area responsible for planning of motoric actions such as scratching [35]–[36]. The lentiform nucleus is considered to modulate the so called cortico-thalamo-cortico circuit that plays a role in planning motor actions like scratching [37], too. So a higher activity of the lentiform nucleus in females could be explained by the higher itch sensation in females that results in a stronger activation of brain structures responsible for scratching compared to males (Table 3).

These findings are particularly based on the strong brain activation during lower leg stimulation in females corresponding to our psychophysical data (Figure 2C). In addition, females also showed an activation of the precentral gyrus (BA 9) and the thalamus during lower leg stimulation (contrast females>males). The precentral gyrus is a well-known structure of sensoric integration of itch [36]–[37]. The thalamus in interplay with the lentiform nucleus plays a role in planning motor actions. These findings are well in line with the significant higher itch intensities at the lower leg in females (Figure 2C). These findings are also supported by our correlation analysis (Tables 8 and 9). A negative correlation of itch intensities with activity strengths of the thalamus, cerebellum and cingulate gyrus were found in males. These structures are well known in the sensoric integration of itch and the planning of motor actions. These negative correlations were not observed in females with our given threshold.

### Central Itch Processing during Distraction

Since both sexes showed comparable performance in the Stroop task (similar reaction times, error rates, Table 2), it is feasible to assume that the observed sex differences in central processing are related to a different central perception but not to performance differences. To our knowledge there are no published studies up-to-date dealing with sex-specific differences during Stroop tasks or distraction in general. Therefore it is difficult to interpret our findings in line with the current literature and one can only speculate.

Interestingly, females reported a significant reduction of itch sensation during the second stimulation at the lower leg (B2) by distraction in contrast to males (Figures 2A and B). These differences are also obvious in brain activation pattern. Here, females presented a higher activation of the posterior cingulate gyrus (BA 29) and a positive correlation of itch ratings with frontal areas activities (including SMA) and the cerebellum in contrast to males. Males, in contrast, depicted a positive correlation with the cerebellum and pre- and postcentral gyri, but a negative correlation with different parts of pre- and postcentral gyri and the cingulate gyrus (Table 9, Figure 5). These findings support our hypothesis that females might have a different distraction strategy, since the observed itch reduction in females is not only associated with brain areas responsible for motor planning but also for emotional integration of stimuli. Therefore it is feasible to assume that females are more emotionally engaged during distraction from itch that may result in a reduced itch sensation. In contrast distraction in males (that is not associated with an itch reduction) seems to provoke only brain regions that are responsible for motoric planning (cerebellum) by the urge to scratch and that seem not to result in lower itch intensity. To summarize, our data point to a different sex-specific correlation pattern indicating that males and females may use different brain networks during distraction yielding to different itch reduction efficiencies.

## Conclusions

Women and men exhibited differences in their itch perception as reflected in higher itch intensities and desire to scratch in women. Itch intensity can be reduced more efficiently by distraction in the lower legs in women, while it can be reduced more efficiently in the forearms in men. In functional brain imaging women generally depicted a higher activation of structures responsible for integration of sensory and affective information as well as motor planning during itch when compared to men.

### Limitations

First, for females, we cannot differ between an effect of localisation and a possible wind-up-phenomenon during the second leg run. It might be possible, that during the last run, the threshold was lower because of the repetitive stimulation. However, since males did not show that behavior, it indicates, that this is a sex-specific phenomenon.

Second, the number of 17 versus 16 participants is not very high. It might be possible that the number of subjects was not sufficient enough to detect other more subtle differences.

Third, we do not have a ‘pure’ itch sensation but a certain mixture with pain sensation. The pain sensation might be provoked by the histamine solution that has a certain effect of burning and be related to the known lower pain threshold of women. But since the pain sensation is much lower than the itch sensation, we assume that pain sensation just plays a secondary role.

Fourth, we cannot exclude completely that the shaving of the women’s leg had some influence of our results. We did not ask the women when they shaved their legs the last time and how they did it (shaving, waxing or epilation). But as the itch stimulation was done intradermally a normal shaving could not influence the itch perception because the root of the hair is still present. But as already mentioned we have not documented how many females did waxing or epilation.

## Supporting Information

Table S1 **Mann-Whitney-U-Test of psychophysical data for females and males (NRS) during saline perfusion.**
(DOC)

Table S2 **Main effect of ‘sex’ during histamine perfusion (uncorrected, p<0.001, with a voxel threshold k>47).**
(DOC)

Table S3 **Interaction of ‘sex’ x ‘condition’ (uncorrected, p<0.001, with a voxel threshold k>47).**
(DOC)

Table S4 **Interaction of ‘sex’ x ‘localisation’ (uncorrected, p<0.001, with a voxel threshold k>47).**
(DOC)

Table S5 (A). Sex-specific correlations of itch intensity with brain activity during first forearm stimulation (A1) during ‘itch’ condition (*uncorrected, p<0.001, with a voxel threshold k>47; **FWE corrected, p<0.05, with a voxel threshold k>10). (B). Sex-specific correlations of itch intensity with brain activity during first forearm stimulation (A1) during ‘Stroop’ condition (*uncorrected, p<0.001, with a voxel threshold k>47; **FWE corrected, p<0.05, with a voxel threshold k>10). (C). Sex-specific correlations of itch intensity and brain activation during first lower leg stimulation (B1) during ‘itch’ condition (*uncorrected, p<0.001, with a voxel threshold k>47; **FWE corrected, with a voxel threshold k>10). (D). Sex-specific correlations of itch intensity and brain activation during first lower leg stimulation (B1) during ‘Stroop’ condition (uncorrected, p<0.001, with a voxel threshold k>47). (E). Sex-specific correlations of itch intensity with brain activity during second forearm stimulation (A2) during ‘itch’ condition (uncorrected, p<0.001, with a voxel threshold k>47). (F). Sex-specific correlations of itch intensity with brain activity during second forearm stimulation (A2) during ‘Stroop’ condition (uncorrected, p<0.001, with a voxel threshold k>47).
(DOC)

## References

[1] Gupta MA, Gupta AK, Kirkby S, Weiner HK, Mace TM, et al (1988) Pruritus in psoriasis. A prospective study of some psychiatric and dermatologic correlates. Arch Dermatol 124: 1052–1057. 3389849 10.1001/archderm.124.7.1052

[2] Gupta MA, Gupta AK, Schork NJ, Ellis CN (1994) Depression modulates pruritus perception: a study of pruritus in psoriasis, atopic dermatitis, and chronic idiopathic urticaria. Psychosom Med 56: 36–40. 8197313 10.1097/00006842-199401000-00005

[3] Schneider G, Driesch G, Heuft G, Evers S, Luger TA, et al (2006) Psychosomatic cofactors and psychiatric comorbidity in patients with chronic itch. Clin Exp Dermatol 31: 762–767. 17040260 10.1111/j.1365-2230.2006.02211.x

[4] Mercuro G, Deidda M, Piras A, Dessalvi CC, Maffei S, et al (2010) Gender determinants of cardiovascular risk factors and diseases. J Cardiovasc Med (Hagerstown ) 11: 207–220. 19829128 10.2459/JCM.0b013e32833178ed

[5] Mercuro G, Deidda M, Bina A, Manconi E, Rosano GM (2011) Gender-specific aspects in primary and secondary prevention of cardiovascular disease. Curr Pharm Des 17: 1082–1089. 21449885 10.2174/138161211795656954

[6] Schuler MS, Lechner WV, Carter RE, Malcolm R (2009) Temporal and gender trends in concordance of urine drug screens and self-reported use in cocaine treatment studies. J Addict Med 3: 211–217. 20209029 10.1097/ADM.0b013e3181a0f5dc PMC2832304

[7] Schuler MP, Barclay A, Harrison B, Larson P (1986) Psychological services offered to female veterans. J Clin Psychol 42: 668–675. 3745468 10.1002/1097-4679(198607)42:4<668::aid-jclp2270420424>3.0.co;2-i

[8] Vitale C, Miceli M, Rosano GM (2007) Gender-specific characteristics of atherosclerosis in menopausal women: risk factors, clinical course and strategies for prevention. Climacteric 10 Suppl 216–20. 17882667 10.1080/13697130701602712

[9] Picci RL, Vigna-Taglianti F, Oliva F, Mathis F, Salmaso S, et al (2012) Personality disorders among patients accessing alcohol detoxification treatment: prevalence and gender differences. Compr Psychiatry 53: 355–363. 21821240 10.1016/j.comppsych.2011.05.011

[10] Ständer S, Stumpf A, Osada N, Wilp S, Chatzigeorgakidis E, et al (2013) Gender differences in chronic pruritus: women present different morbidity, more scratch lesions and higher burden. Br J Dermatol 168: 1273–1280. 23387396 10.1111/bjd.12267

[11] Holm EA, Esmann S, Jemec GB (2004) Does visible atopic dermatitis affect quality of life more in women than in men? Gend Med 1: 125–130. 16115590 10.1016/s1550-8579(04)80017-2

[12] Uttjek M, Dufaker M, Nygren L, Stenberg B (2005) Psoriasis care consumption and expectations from a gender perspective in a psoriasis population in northern Sweden. Acta Derm Venereol 85: 503–508. 16396797 10.1080/00015550510036667

[13] Drzezga A, Darsow U, Treede RD, Siebner H, Frisch M, et al (2001) Central activation by histamine-induced itch: analogies to pain processing: a correlational analysis of O-15 H2O positron emission tomography studies. Pain 92: 295–305. 11323151 10.1016/s0304-3959(01)00271-8

[14] Ständer S, Schmelz M (2006) Chronic itch and pain–similarities and differences. Eur J Pain 10: 473–478. 16678456 10.1016/j.ejpain.2006.03.005

[15] Henderson LA, Gandevia SC, Macefield VG (2008) Gender differences in brain activity evoked by muscle and cutaneous pain: a retrospective study of single-trial fMRI data. Neuroimage 39: 1867–1876. 18069004 10.1016/j.neuroimage.2007.10.045

[16] Paulson PE, Minoshima S, Morrow TJ, Casey KL (1998) Gender differences in pain perception and patterns of cerebral activation during noxious heat stimulation in humans. Pain 76: 223–229. 9696477 10.1016/s0304-3959(98)00048-7 PMC1828033

[17] Derbyshire SW, Nichols TE, Firestone L, Townsend DW, Jones AK (2002) Gender differences in patterns of cerebral activation during equal experience of painful laser stimulation. J Pain 3: 401–411. 14622744 10.1054/jpai.2002.126788

[18] Bantick SJ, Wise RG, Ploghaus A, Clare S, Smith SM, et al (2002) Imaging how attention modulates pain in humans using functional MRI. Brain 125: 310–319. 11844731 10.1093/brain/awf022

[19] Eccleston C (1995) Chronic pain and distraction: an experimental investigation into the role of sustained and shifting attention in the processing of chronic persistent pain. Behav Res Ther 33: 391–405. 7538753 10.1016/0005-7967(94)00057-q

[20] Leibovici V, Magora F, Cohen S, Ingber A (2009) Effects of virtual reality immersion and audiovisual distraction techniques for patients with pruritus. Pain Res Manag 14: 283–286. 19714267 10.1155/2009/178751 PMC2734514

[21] Valet M, Sprenger T, Boecker H, Willoch F, Rummeny E, et al (2004) Distraction modulates connectivity of the cingulo-frontal cortex and the midbrain during pain–an fMRI analysis. Pain 109: 399–408. 15157701 10.1016/j.pain.2004.02.033

[22] Keogh E, Eccleston C (2006) Sex differences in adolescent chronic pain and pain-related coping. Pain 123: 275–284. 16644131 10.1016/j.pain.2006.03.004

[23] Unrod M, Kassel JD, Robinson M (2004) Effects of smoking, distraction, and gender on pain perception. Behav Med 30: 133–139. 15816316 10.3200/BMED.30.3.133-140

[24] Touyz LZ, Lamontagne P, Smith BE (2004) Pain and anxiety reduction using a manual stimulation distraction device when administering local analgesia oro-dental injections: a multi-center clinical investigation. J Clin Dent 15: 88–92. 15688965

[25] Bradley MM, Lang PJ (1994) Measuring emotion: the Self-Assessment Manikin and the Semantic Differential. J Behav Ther Exp Psychiatry 25: 49–59. 7962581 10.1016/0005-7916(94)90063-9

[26] Friston KJ, Ashburner J, Kiebel SJ, Nichols TE, Penny WD (2007) Statistical Parametric Mapping. The Analysis of Functional Brain Images.

[27] Slotnick SD, Moo LR, Segal JB, Hart J Jr (2003) Distinct prefrontal cortex activity associated with item memory and source memory for visual shapes. Brain Res Cogn Brain Res 17: 75–82. 12763194 10.1016/s0926-6410(03)00082-x

[28] Magerl W, Westerman RA, Mohner B, Handwerker HO (1990) Properties of transdermal histamine iontophoresis: differential effects of season, gender, and body region. J Invest Dermatol 94: 347–352. 2307854 10.1111/1523-1747.ep12874474

[29] Fillingim RB, King CD, Ribeiro-Dasilva MC, Rahim-Williams B, Riley JL III (2009) Sex, gender, and pain: a review of recent clinical and experimental findings. J Pain 10: 447–485. 19411059 10.1016/j.jpain.2008.12.001 PMC2677686

[30] Lautenbacher S, Rollman GB (1993) Sex differences in responsiveness to painful and non-painful stimuli are dependent upon the stimulation method. Pain 53: 255–264. 8351155 10.1016/0304-3959(93)90221-A

[31] Truini A, Leone C, Di SG, Biasiotta A, La CS, et al (2011) Topographical distribution of warmth, burning and itch sensations in healthy humans. Neurosci Lett 494: 165–168. 21396428 10.1016/j.neulet.2011.03.004

[32] Bergeret L, Black D, Theunis J, Misery L, Chauveau N, et al (2011) Validation of a model of itch induction for brain positron emission tomography studies using histamine iontophoresis. Acta Derm Venereol 91: 504–510. 21874218 10.2340/00015555-1067

[33] Lorenz J, Minoshima S, Casey KL (2003) Keeping pain out of mind: the role of the dorsolateral prefrontal cortex in pain modulation. Brain 126: 1079–1091. 12690048 10.1093/brain/awg102

[34] Benson S, Kotsis V, Rosenberger C, Bingel U, Forsting M, et al (2012) Behavioural and neural correlates of visceral pain sensitivity in healthy men and women: does sex matter? Eur J Pain 16: 349–358. 22337318 10.1002/j.1532-2149.2011.00027.x

[35] Papoiu AD, Coghill RC, Kraft RA, Wang H, Yosipovitch G (2012) A tale of two itches. Common features and notable differences in brain activation evoked by cowhage and histamine induced itch. Neuroimage 59: 3611–3623. 22100770 10.1016/j.neuroimage.2011.10.099 PMC3288667

[36] Kleyn CE, McKie S, Ross A, Elliott R, Griffiths CE (2012) A temporal analysis of the central neural processing of itch. Br J Dermatol 166: 994–1001. 22283926 10.1111/j.1365-2133.2012.10849.x

[37] Schneider G, Ständer S, Burgmer M, Driesch G, Heuft G, et al (2008) Significant differences in central imaging of histamine-induced itch between atopic dermatitis and healthy subjects. Eur J Pain 12: 834–841. 18203636 10.1016/j.ejpain.2007.12.003

