# Supplementary material for: Sex Differences in Itch Perception and Modulation by Distraction – an fMRI Pilot Study in Healthy Volunteers
Source: PLoS One. 2013 Nov 18;8(11):e79123. doi: 10.1371/journal.pone.0079123 (PMC3832610; doi:10.1371/journal.pone.0079123)
Supplement: Table S1 — Mann-Whitney-U-Test of psychophysical data for females and males (NRS) during saline perfusion. (DOC) [file pone.0079123.s001.doc]

**Table S1. Mann-Whitney-U-Test of psychophysical data for females and males (NRS) during saline perfusion.**

|  | **Females** | | **Males** | | **p** |
| --- | --- | --- | --- | --- | --- |
|  | Mean | SD | Mean | SD |  |
| Itch sensation | | | | | |
| A1_J* | 0.8 | 1.8 | 0.4 | 0.5 | n.s. |
| A1_S* | 0.4 | 0.5 | 0.4 | 0.9 | n.s. |
| B1_S* | 0.6 | 0.5 | 0.6 | 0.5 | n.s. |
| B1_J* | 0.2 | 0.4 | 0.0 | 0.0 | n.s. |
| A2_S* | 0.0 | 0.0 | 0.0 | 0.0 | n.s. |
| A2_J* | 0.2 | 0.4 | 0.0 | 0.0 | n.s. |
| B2_J* | 1.0 | 1.7 | 0.6 | 0.9 | n.s. |
| B2_S* | 0.4 | 0.5 | 0.2 | 0.4 | n.s. |
| Desire to scratch | | | | | |
| A1_J* | 0.4 | 0.5 | 0.0 | 0.0 | n.s. |
| A1_S* | 0.2 | 0.4 | 0.2 | 0.4 | n.s. |
| B1_S* | 0.4 | 0.5 | 0.0 | 0.0 | n.s. |
| B1_J* | 0.0 | 0.0 | 0.0 | 0.0 | n.s. |
| A2_S* | 0.4 | 0.9 | 0.2 | 0.4 | n.s. |
| A2_J* | 0.2 | 0.4 | 0.2 | 0.4 | n.s. |
| B2_J* | 0.8 | 1.3 | 0.4 | 0.5 | n.s. |
| B2_S* | 0.2 | 0.4 | 0.0 | 0.0 | n.s. |
| Pain sensation | | | | | |
| A1_J* | 0.8 | 1.3 | 1.0 | 1.0 | n.s. |
| A1_S* | 0.4 | 0.5 | 0.6 | 0.9 | n.s. |
| B1_S* | 0.2 | 0.4 | 0.6 | 0.9 | n.s. |
| B1_J* | 0.4 | 0.5 | 0.8 | 0.8 | n.s. |
| A2_S* | 1.0 | 1.7 | 1.2 | 1.1 | n.s. |
| A2_J* | 1.8 | 3.5 | 1.4 | 0.9 | n.s. |
| B2_J* | 0.8 | 0.8 | 1.6 | 1.7 | n.s. |
| B2_S* | 0.8 | 0.8 | 0.6 | 1.3 | n.s. |

^*)^ A1_J = first run forearm during ‘itch’, A1_S = first run forearm during ‘Stroop’, B1_J = first run lower leg during ‘itch’, B1_S = first run lower leg during ‘Stroop’, A2_J = second run forearm during ‘itch’, A2_S = second run forearm during ‘Stroop’, B2_J = second run lower leg during ‘itch’, B2_S = second run lower leg during ‘Stroop’
