# Supplementary material for: Sex Differences in Itch Perception and Modulation by Distraction – an fMRI Pilot Study in Healthy Volunteers
Source: PLoS One. 2013 Nov 18;8(11):e79123. doi: 10.1371/journal.pone.0079123 (PMC3832610; doi:10.1371/journal.pone.0079123)
Supplement: Table S2 — Main effect of ‘sex’ during histamine perfusion (uncorrected, p<0.001, with a voxel threshold k>47). (DOC) [file pone.0079123.s002.doc]

**Table S2. Main effect of ‘sex’ during histamine perfusion (uncorrected, p < 0.001, with a voxel threshold k > 47).**

| Region | k | Z-score | p (uncorr.) | coordinates (x y z mm) | | |
| --- | --- | --- | --- | --- | --- | --- |
| Left cuneus (BA 18) | 103 | 5.32 | <0.0001 | -14 | -102 | 4 |
| Left inf. frontal gyrus (BA 47)* | 1031 | 4.87 | <0.0001 | -48 | 22 | -14 |
|  |  | 4.74 | <0.0001 | -40 | 20 | -12 |
| Left ant. cingulate gyrus (BA 10)* |  | 3.74 | <0.0001 | -16 | 28 | -6 |
| Right middle frontal gyrus (BA 8) | 326 | 4.72 | <0.0001 | 40 | 38 | 38 |
| Right middle frontal gyrus; DLPFC (BA 9)* |  | 4.17 | <0.0001 | 36 | 26 | 34 |
| Left medial frontal gyrus (BA 9) | 939 | 4.69 | <0.0001 | 0 | 36 | 34 |
| Right medial frontal gyrus (BA 8) |  | 4.25 | <0.0001 | 10 | 30 | 42 |
| Right inf. frontal gyrus (BA 47) | 587 | 4.49 | <0.0001 | 54 | 20 | -2 |
|  |  | 4.08 | <0.0001 | 48 | 26 | -4 |
| Right inf. frontal gyrus (BA 45) |  | 3.73 | <0.0001 | 36 | 24 | 6 |
| Right inf. parietal lobule (BA 40) | 231 | 4.31 | <0.0001 | 56 | -46 | 52 |
|  |  | 3.98 | <0.0001 | 50 | -54 | 54 |
|  |  | 3.22 | 0.001 | 64 | -30 | 42 |
| Right caudate body | 94 | 4.24 | <0.0001 | 20 | 20 | 8 |
| Right lentiform nucleus |  | 3.12 | 0.001 | 24 | 10 | 10 |
| Right sup. frontal gyrus; SMA (BA 6)* | 104 | 4.22 | <0.0001 | 4 | 20 | 62 |
| Left inf. parietal lobule (BA 40)* | 186 | 4.08 | <0.0001 | -50 | -54 | 52 |
|  |  | 3.31 | <0.0001 | -36 | -52 | 40 |
| Left cerebellum | 76 | 3.86 | <0.0001 | -28 | -68 | -30 |
|  |  | 3.39 | <0.0001 | -30 | -60 | -32 |
| Left sup. frontal gyrus (BA 9)* | 52 | 3.79 | <0.0001 | -26 | 50 | 38 |
| Right sup. frontal gyrus (BA 10)* | 68 | 3.69 | <0.0001 | 30 | 56 | 14 |
| Left caudate head | 79 | 3.69 | <0.0001 | -18 | 20 | 6 |

^*)^ inf. = inferior, sup. = superior, ant. = anterior, DLPFC = dorsolateral prefrontal cortex, SMA = supplementary motor area
