# Supplementary material for: Sex Differences in Itch Perception and Modulation by Distraction – an fMRI Pilot Study in Healthy Volunteers
Source: PLoS One. 2013 Nov 18;8(11):e79123. doi: 10.1371/journal.pone.0079123 (PMC3832610; doi:10.1371/journal.pone.0079123)
Supplement: Table S3 — Interaction of ‘sex’ x ‘condition’ (uncorrected, p<0.001, with a voxel threshold k>47). (DOC) [file pone.0079123.s003.doc]

**Table S3. Interaction of ‘sex’ x ‘condition’ (uncorrected, p < 0.001, with a voxel threshold k > 47).**

| Region | k | Z-score | p (uncorr.) | coordinates (x y z mm) | | |
| --- | --- | --- | --- | --- | --- | --- |
| Left middle temporal gyrus (BA 21) | 73 | 3.84 | <0.0001 | -54 | 6 | -32 |
| Left middle temporal gyrus (BA 38) |  | 3.72 | <0.0001 | -46 | 4 | -40 |
|  |  | 3.32 | <0.0001 | -38 | 12 | -42 |
| Left lingual gyrus (BA 18) | 105 | 3.69 | <0.0001 | -12 | -64 | -2 |
| Left posterior cingulate gyrus (BA 30) |  | 3.43 | <0.0001 | -16 | -58 | 4 |
| Right posterior cingulate gyrus (BA 30) | 110 | 3.61 | <0.0001 | 2 | -62 | 8 |
| Right cerebellum |  | 3.26 | 0.001 | 12 | -60 | -6 |
| Left cuneus (BA 19) | 116 | 3.58 | <0.0001 | -4 | -90 | 36 |
| Left middle occipital gyrus (BA 18) |  | 3.5 | <0.0001 | -8 | -94 | 16 |
| Left cuneus (BA 19) |  | 3.48 | <0.0001 | -2 | -90 | 26 |
