# Supplementary material for: Sex Differences in Itch Perception and Modulation by Distraction – an fMRI Pilot Study in Healthy Volunteers
Source: PLoS One. 2013 Nov 18;8(11):e79123. doi: 10.1371/journal.pone.0079123 (PMC3832610; doi:10.1371/journal.pone.0079123)
Supplement: Table S4 — Interaction of ‘sex’ x ‘localisation’ (uncorrected, p<0.001, with a voxel threshold k>47). (DOC) [file pone.0079123.s004.doc]

**Table S4. Interaction of ‘sex’ x ‘localisation’ (uncorrected, p < 0.001, with a voxel threshold k > 47).**

| Region | k | Z-score | p (uncorr.) | coordinates (x y z mm) | | |
| --- | --- | --- | --- | --- | --- | --- |
| Right precentral gyrus (BA 6) | 173 | 5.4 | <0.0001 | 30 | 0 | 34 |
| Right lingual gyrus | 131 | 4.93 | <0.0001 | 30 | -72 | 2 |
| Right thalamus | 113 | 4.15 | <0.0001 | 24 | -26 | 14 |
| Right insula | 66 | 3.49 | <0.0001 | 36 | -46 | 20 |
