# Supplementary material for: Sex Differences in Itch Perception and Modulation by Distraction – an fMRI Pilot Study in Healthy Volunteers
Source: PLoS One. 2013 Nov 18;8(11):e79123. doi: 10.1371/journal.pone.0079123 (PMC3832610; doi:10.1371/journal.pone.0079123)
Supplement: Table S5 — (A). Sex-specific correlations of itch intensity with brain activity during first forearm stimulation (A1) during ‘itch’ condition (*uncorrected, p<0.001, with a voxel threshold k>47; **FWE corrected, p<0.05, with a voxel threshold k>10). (B). Sex-specific correlations of itch intensity with brain activity during first forearm stimulation (A1) during ‘Stroop’ condition (*uncorrected, p<0.001, with a voxel threshold k>47; **FWE corrected, p<0.05, with a voxel threshold k>10). (C). Sex-specific correlations of itch intensity and brain activation during first lower leg stimulation (B1) during ‘itch’ condition (*uncorrected, p<0.001, with a voxel threshold k>47; **FWE corrected, with a voxel threshold k>10). (D). Sex-specific correlations of itch intensity and brain activation during first lower leg stimulation (B1) during ‘Stroop’ condition (uncorrected, p<0.001, with a voxel threshold k>47). (E). Sex-specific correlations of itch intensity with brain activity during second forearm stimulation (A2) during ‘itch’ condition (uncorrected, p<0.001, with a voxel threshold k>47). (F). Sex-specific correlations of itch intensity with brain activity during second forearm stimulation (A2) during ‘Stroop’ condition (uncorrected, p<0.001, with a voxel threshold k>47). (DOC) [file pone.0079123.s005.doc]

**Table S5A. Sex-specific correlations of itch intensity with brain activity during first forearm stimulation (A1) during ‘itch’ condition (* uncorrected, p < 0.001, with a voxel threshold k > 47; ** FWE corrected, p < 0.05, with a voxel threshold k > 10).**

| Region | k | Z-score | p | coordinates (x y z mm) | | | | | | |
| --- | --- | --- | --- | --- | --- | --- | --- | --- | --- | --- |
| **females** | | | | | | | | | | |
| *positive correlation** | | | | | | | | | | |
| Left insula | 225 | 5.10 | <0.0001 | -40 | | | | -18 | | 20 |
| Left claustrum |  | 3.72 | <0.0001 | -32 | | | | -16 | | 8 |
| Left insula |  | 3.46 | <0.0001 | -46 | | | | -10 | | 14 |
| Left medial frontal gyrus (BA 11) | 136 | 4.23 | <0.0001 | -6 | | | | 52 | | -10 |
| Left medial frontal gyrus (BA 10) |  | 3.77 | <0.0001 | 0 | | | | 60 | | -10 |
|  |  | 3.29 | 0.001 | -2 | | | | 58 | | -2 |
| Right insula | 642 | 4.14 | <0.0001 | 42 | | | | -14 | | -2 |
|  |  | 3.94 | <0.0001 | 38 | | | | -36 | | 18 |
|  |  | 3.84 | <0.0001 | 46 | | | | -10 | | 14 |
| Right medial frontal gyrus (BA 6) | 59 | 3.89 | <0.0001 | 8 | | | | -18 | | 54 |
| Left paracentral lobule (BA 5) | 173 | 3.89 | <0.0001 | -16 | | | | -34 | | 46 |
| Left precuneus (BA 7) |  | 3.82 | <0.0001 | -6 | | | | -32 | | 44 |
| Right paracentral lobule (BA 6) |  | 3.26 | 0.001 | 6 | | | | -30 | | 54 |
| Left angular gyrus (BA 39) | 67 | 3.75 | <0.0001 | -52 | | | | -72 | | 32 |
| Left superior temporal gyrus (BA 22) | 51 | 3.61 | <0.0001 | -52 | | | | -6 | | 2 |
| Right postcentral gyrus (BA 43) | 149 | 3.61 | <0.0001 | 66 | | | | -6 | | 16 |
| Right transverse temporal gyrus (BA 42) | 225 | 3.58 | <0.0001 | 58 | | | | -14 | | 10 |
| *Negative correlation*** | | | | | | | | | | |
| Left middle occipital gyrus (BA 19) | 108 | 5.75 | 0.001 | -30 | | | -82 | | | 20 |
|  |  | 5.29 | 0.009 | -30 | | | -90 | | | 14 |
|  | 359 | 5.64 | 0.002 | -30 | | | -98 | | | 0 |
| Left fusiform gyrus (BA 18) |  | 5.43 | 0.005 | -28 | | | -92 | | | -16 |
| Left fusiform gyrus (BA 19) |  | 5.28 | 0.009 | -38 | | | -70 | | | -12 |
| Left cerebellum | 17 | 5.48 | 0.004 | -36 | | | -78 | | | -28 |
|  | 72 | 5.46 | 0.004 | -34 | | | -88 | | | -16 |
| Left lingual gyrus (BA 17) |  | 5.40 | 0.006 | 22 | | | -98 | | | -12 |
| Left precuneus (BA 7) | 40 | 5.45 | 0.004 | -22 | | | -64 | | | 32 |
| Right middle occipital gyrus (BA 18) | 94 | 5.45 | 0.005 | 36 | | | -88 | | | 0 |
|  |  | 5.37 | 0.007 | 36 | | | -86 | | | 12 |
| Left inferior occipital gyrus (BA 19) | 13 | 5.40 | 0.006 | -42 | | | -80 | | | 0 |
| Right cerebellum | 52 | 5.30 | 0.009 | 38 | | | -74 | | | -18 |
|  |  | 4.96 | 0.034 | 42 | | | -66 | | | -18 |
| Right precuneus (BA 7) | 49 | 5.27 | 0.010 | 28 | | | -70 | | | 48 |
| **males** | | | | | | | | | | |
| *positive correlation** | | | | | | | | | | |
| Right postcentral gyrus (BA 3) | 2616 | 5.10 | <0.0001 | 20 | | | -30 | | | 60 |
| Left precentral gyrus (BA 6) |  | 4.62 | <0.0001 | -10 | | | -18 | | | 60 |
| Right medial frontal gyrus (BA 6) |  | 4.59 | <0.0001 | 12 | | | -14 | | | 60 |
| Left precuneus (BA 31) | 887 | 4.52 | <0.0001 | -6 | | | -62 | | | 24 |
| Left posterior cingulate gyrus (BA 23) |  | 4.34 | <0.0001 | -2 | -58 | | | | | 18 |
| Right posterior cingulate gyrus (BA 29) |  | 4.29 | <0.0001 | 10 | -48 | | | | | 14 |
| Right precentral gyrus (BA 4) | 262 | 4.09 | <0.0001 | 44 | | -12 | | | | 42 |
| Right postcentral gyrus (BA 3) |  | 3.84 | <0.0001 | 46 | | -22 | | | | 62 |
|  |  | 3.57 | <0.0001 | 42 | | -16 | | | | 52 |
| Left precentral gyrus (BA 6) | 112 | 4.07 | <0.0001 | -64 | | -4 | | | | 26 |
| Left postcentral gyrus (BA 43) |  | 3.73 | <0.0001 | -64 | | -4 | | | | 14 |
|  |  | 3.34 | <0.0001 | -52 | | -6 | | | | 18 |
| Right superior temporal gyrus (BA 22) | 54 | 3.94 | <0.0001 | 52 | | -10 | | | | -10 |
| Right middle temporal gyrus (BA 21) |  | 3.81 | <0.0001 | 64 | | -10 | | | | -8 |
| Right anterior cingulate gyrus (BA 32) | 63 | 3.93 | <0.0001 | 18 | | 40 | | | | 0 |
| Right insula | 332 | 3.84 | <0.0001 | 46 | | -12 | | | | 14 |
| Right superior temporal gyrus (BA 22) |  | 3.82 | <0.0001 | 56 | | -6 | | | | 8 |
| Right insula |  | 3.52 | <0.0001 | 38 | | -10 | | | | 10 |
| Left superior occipital gyrus (BA 19) | 54 | 3.71 | <0.0001 | -44 | | -82 | | | | 32 |
| Left middle temporal gyrus (BA 39) |  | 3.44 | <0.0001 | -50 | | -78 | | | | 26 |
|  |  | 3.38 | <0.0001 | -52 | | -72 | | | | 18 |
| Left medial frontal gyrus (BA 10) | 111 | 3.69 | <0.0001 | -4 | | 42 | | | | -8 |
| *negative correlation*** | | | | | | | | | | |
| Left inferior occipital gyrus (BA 18) | 94 | 5.56 | 0.003 | -34 | | | | | -94 | -14 |
|  |  | 5.44 | 0.005 | -30 | | | | | -94 | -4 |
| Right cerebellum | 13 | 5.48 | 0.004 | 42 | | | | | -60 | -16 |
| Right lingual gyrus (BA 17) | 11 | 5.41 | 0.006 | 22 | | | | | -100 | -10 |
| Left lingual gyrus (BA 17) | 14 | 5.29 | 0.012 | -20 | | | | | -102 | -10 |
| Left cerebellum | 30 | 5.21 | 0.016 | -34 | | | | | -64 | -30 |

**Table S5B. Sex-specific correlations of itch intensity with brain activity during first forearm stimulation (A1) during ‘Stroop’ condition (* uncorrected, p < 0.001, with a voxel threshold k > 47; ** FWE corrected, p < 0.05, with a voxel threshold k > 10).**

| Region | k | | Z-score | p | coordinates (x y z mm) | | | | | | |
| --- | --- | --- | --- | --- | --- | --- | --- | --- | --- | --- | --- |
| **females** | | | | | | | | | | | |
| *positive correlation*** | | | | | | | | | | | |
| Left precentral gyrus (BA 6) | 224 | | 5.93 | <0.0001 | -40 | | | | -16 | | 58 |
|  |  | | 5.87 | <0.0001 | -28 | | | | -18 | | 64 |
|  |  | | 5.23 | 0.009 | -28 | | | | -18 | | 54 |
| Right cuneus (BA 18) | 179 | | 5.64 | 0.001 | 16 | | | | -96 | | 18 |
| Right middle occipital gyrus (BA 19) |  | | 5.19 | 0.010 | 28 | | | | -84 | | 14 |
| Left cuneus (BA 18) |  | | 5.03 | 0.020 | 0 | | | | -98 | | 18 |
|  | 87 | | 5.57 | 0.002 | -14 | | | | -94 | | 24 |
| Right cerebellum | 52 | | 5.31 | 0.006 | 18 | | | | -80 | | -22 |
| Left cuneus (BA 18) | 19 | | 5.27 | 0.007 | -10 | | | | -102 | | 10 |
| Right middle occipital gyrus (BA 19) | 15 | | 5.01 | 0.021 | 48 | | | | -76 | | -6 |
| *Negative correlation** | | | | | | | | | | | |
| no threshold clusters |  | |  |  |  | |  | | | |  |
| **males** | | | | | | | | | | | |
| *positive correlation*** | | | | | | | | | | | |
| Right middle occipital gyrus (BA 18) | | 349 | 6.56 | <0.0001 | | 20 | | | -90 | | 12 |
| Right cuneus (BA 18) | |  | 5.65 | 0.002 | | 10 | | | -94 | | 10 |
| Left middle occipital gyrus (BA 18) | | 120 | 6.16 | <0.0001 | | -18 | | | -96 | | 8 |
| Left middle occipital gyrus (BA 19) | |  | 5.62 | 0.002 | | -26 | | | -88 | | 6 |
| Right cerebellum | | 200 | 5.81 | 0.001 | | 34 | | | -62 | | -6 |
| Right fusiform gyrus (BA 37) | |  | 5.66 | 0.001 | | 40 | | | -54 | | -10 |
| Right parahippocampal gyrus (BA 36) | |  | 5.66 | 0.001 | | 22 | | -44 | | | -8 |
| Left inferior occipital gyrus (BA 19) | | 29 | 5.76 | 0.001 | | -36 | | -74 | | | -8 |
| Right fusiform gyrus (BA 18) | | 51 | 5.53 | 0.003 | | 20 | | -90 | | | -14 |
| Right lingual gyrus (BA 18) | |  | 5.08 | 0.031 | | 6 | | -84 | | | -4 |
| Right fusiform gyrus (BA 37) | | 17 | 5.52 | 0.003 | | 44 | | -44 | | | -12 |
| *negative correlation** | | | | | | | | | | | |
| Right cuneus (BA 7) | | 235 | 4.41 | <0.0001 | 14 | | | | | -68 | 32 |
| Left posterior cingulate gyrus (BA 23) | | 92 | 3.70 | <0.0001 | -8 | | | | | -30 | 26 |
|  | |  | 3.44 | <0.0001 | -6 | | | | | -22 | 26 |
| Right posterior cingulate gyrus (BA 23) | |  | 3.23 | 0.001 | 8 | | | | | -28 | 26 |
| Left precuneus (BA 7) | | 65 | 3.49 | <0.0001 | -8 | | | | | -74 | 34 |
|  | |  | 3.32 | <0.0001 | -12 | | | | | -66 | 32 |

**Table S5C. Sex-specific correlations of itch intensity and brain activation during first lower leg stimulation (B1) during ‘itch’ condition (* uncorrected, p < 0.001, with a voxel threshold k > 47; ** FWE corrected, with a voxel threshold k > 10).**

| Region | k | Z-score | p | coordinates (x y z mm) | | | |
| --- | --- | --- | --- | --- | --- | --- | --- |
| **females** | | | | | | | |
| *positive correlation** | | | | | | | |
| Left insula | 964 | 5.19 | <0.0001 | -34 | -18 | 20 | |
| Left middle temporal gyrus (BA 21) |  | 4.34 | <0.0001 | -62 | -10 | -12 | |
| Left insula |  | 4.16 | <0.0001 | -40 | -6 | -2 | |
| Left fusiform gyrus (BA 20) | 2546 | 4.91 | <0.0001 | -36 | -34 | -18 | |
| Left cuneus (BA 30) |  | 4.24 | <0.0001 | -20 | -68 | 12 | |
| Left lingual gyrus (BA 18) |  | 4.24 | <0.0001 | -10 | -58 | 6 | |
| Right lingual gyrus (BA18) | 73 | 4.83 | <0.0001 | 4 | -86 | -8 | |
| Left precentral gyrus (BA 4) | 62 | 4.71 | <0.0001 | -52 | -2 | 16 | |
| Left superior temporal gyrus (BA 22) |  | 3.30 | <0.0001 | -56 | 0 | 6 | |
| Right fusiform gyrus (BA 20) | 196 | 4.68 | <0.0001 | 38 | -30 | -16 | |
| Right parahippocampal gyrus (BA 36) |  | 4.10 | <0.0001 | 28 | -18 | -22 | |
|  |  | 3.89 | <0.0001 | 36 | -22 | -18 | |
| Right insula | 709 | 4.64 | <0.0001 | 40 | -28 | 20 | |
|  |  | 4.00 | <0.0001 | 40 | -18 | 14 | |
| Right precentral gyrus (BA 6) |  | 3.97 | <0.0001 | 48 | -8 | 10 | |
| Left medial frontal gyrus (BA 11) | 1391 | 4.59 | <0.0001 | -4 | 56 | -14 | |
| Right medial frontal gyrus (BA 10) |  | 4.51 | <0.0001 | 2 | 58 | -4 | |
| Left anterior cingulate gyrus (BA 32) |  | 4.43 | <0.0001 | -4 | 40 | -6 | |
| Right postcentral gyrus (BA 3) | 407 | 4.32 | <0.0001 | 44 | -24 | 66 | |
| Right postcentral gyrus (BA 2) |  | 4.13 | <0.0001 | 36 | -38 | 70 | |
| Right postcentral gyrus (BA 3) |  | 3.57 | <0.0001 | 38 | -26 | 52 | |
| Right superior temporal gyrus (BA 38) | 51 | 3.87 | <0.0001 | 38 | 12 | -24 | |
| Left middle temporal gyrus (BA 39) | 143 | 3.85 | <0.0001 | -52 | -74 | 24 | |
| Left angular gyrus (BA 39) |  | 3.71 | <0.0001 | -46 | -76 | 32 | |
| Left superior occipital gyrus (BA 19) |  | 3.60 | <0.0001 | -42 | -80 | 38 | |
| Right parahippocampal gyrus (BA 19) | 93 | 3.83 | <0.0001 | 18 | -44 | -4 | |
| right parahippocampal gyrus (BA 30) |  | 3.80 | <0.0001 | 22 | -48 | 2 | |
| Right cerebellum |  | 3.29 | <0.0001 | 18 | -40 | -14 | |
| Right thalamus |  | 3.81 | <0.0001 | 2 | -12 | -2 | |
| Left parahippocampal gyrus (BA 36) | 58 | 3.70 | <0.0001 | -30 | -18 | -24 | |
|  |  | 3.57 | <0.0001 | -26 | -12 | -18 | |
| Left uncus |  | 3.51 | <0.0001 | -20 | -6 | -20 | |
| Left precentral gyrus (BA 4) | 76 | 3.50 | <0.0001 | -12 | -32 | 66 | |
| Left postcentral gyrus (BA 3) |  | 3.49 | <0.0001 | -20 | -34 | 64 | |
| *Negative correlation** | | | | | | | |
| Right superior parietal lobule (BA 7) | 3456 | 5.37 | <0.0001 | 30 | -72 | | 46 |
| Right precuneus (BA 7) |  | 5.07 | <0.0001 | 24 | -74 | | 52 |
| Right middle occipital gyrus (BA 19) |  | 4.85 | <0.0001 | 34 | -88 | | 14 |
| Left cuneus (BA 18) | 2521 | 4.94 | <0.0001 | -28 | -100 | | 2 |
| Left middle occipital gyrus (BA 19) |  | 4.84 | <0.0001 | -30 | -92 | | 14 |
| Left cerebellum |  | 4.84 | <0.0001 | -40 | -74 | | -20 |
| Left precuneus (BA 7) | 735 | 4.47 | <0.0001 | -26 | -58 | | 54 |
| Left precuneus (BA 19) |  | 3.64 | <0.0001 | -24 | -70 | | 36 |
| Left superior parietal lobule (BA 7) |  | 3.51 | <0.0001 | -14 | -66 | | 66 |
| Left cerebellum | 327 | 4.30 | <0.0001 | -2 | -80 | | -32 |
| Right cerebellum |  | 3.83 | <0.0001 | 8 | -80 | | -40 |
|  |  | 3.54 | <0.0001 | 6 | -68 | | -42 |
|  | 165 | 4.16 | <0.0001 | 26 | -54 | | -26 |
|  |  | 4.01 | <0.0001 | 32 | -52 | | -32 |
| **males** | | | | | | | |
| *positive correlation** | | | | | | | |
| Right precentral gyrus (BA 4) | 132 | 3.85 | <0.0001 | 30 | -20 | | 48 |
| Right postcentral gyrus (BA 3) |  | 3.75 | <0.0001 | 52 | -12 | | 58 |
| Right precentral gyrus (BA 4) |  | 3.35 | <0.0001 | 40 | -16 | | 48 |
| Right medial frontal gyrus (BA 6) | 58 | 3.75 | <0.0001 | 14 | -20 | | 54 |
|  |  | 3.25 | 0.001 | 18 | -16 | | 48 |
| Left paracentral lobule (BA 6) | 54 | 3.73 | <0.0001 | -10 | -24 | | 52 |
| *negative correlation*** | | | | | | | |
| Left lingual gyrus (BA 17) | 38 | 5.85 | <0.0001 | -20 | -102 | | -14 |
| Left fusiform gyrus (BA 18) |  | 5.21 | 0.011 | -30 | -96 | | -14 |
| Right lingual gyrus (BA 17) | 17 | 5.36 | 0.006 | 22 | -98 | | -12 |

**Table S5D. Sex-specific correlations of itch intensity and brain activation during first lower leg stimulation (B1) during ‘Stroop’ condition (uncorrected, p < 0.001, with a voxel threshold k > 47).**

| Region | k | Z-score | p | coordinates (x y z mm) | | |
| --- | --- | --- | --- | --- | --- | --- |
| **females** | | | | | | |
| *positive correlation* | | | | | | |
| Left middle occipital gyrus (BA 18) | 256 | 5.71 | <0.0001 | -8 | -100 | 16 |
| Left cuneus (BA 19) |  | 4.39 | <0.0001 | -14 | -96 | 24 |
|  |  | 3.88 | <0.0001 | -8 | -98 | 30 |
| Right cuneus (BA 18) | 466 | 4.68 | <0.0001 | 18 | -90 | 20 |
|  |  | 4.45 | <0.0001 | 12 | -96 | 18 |
| Left cerebellum | 2278 | 4.49 | <0.0001 | -20 | -62 | -18 |
| Left inferior occipital gyrus (BA 19) |  | 4.39 | <0.0001 | -40 | -78 | -6 |
| Left middle occipital gyrus (BA 19) |  | 4.26 | <0.0001 | -46 | -80 | 2 |
| Right inferior occipital gyrus (BA 19) | 478 | 3.91 | <0.0001 | 36 | -78 | -6 |
| Right lingual gyrus (BA 18) |  | 3.78 | <0.0001 | 24 | -80 | -6 |
| Right fusiform gyrus (BA 19) |  | 3.73 | <0.0001 | 36 | -80 | -14 |
| *Negative correlation* | | | | | | |
| Left inferior frontal gyrus (BA 47) | 112 | 4.37 | <0.0001 | -54 | 26 | -4 |
| Left inferior frontal gyrus (BA 45) |  | 3.29 | <0.0001 | -44 | 22 | 4 |
| Left middle temporal gyrus (BA 21) | 167 | 4.30 | <0.0001 | -56 | -32 | -2 |
|  |  | 3.42 | <0.0001 | -66 | -48 | -2 |
| **males** | | | | | | |
| *positive correlation* | | | | | | |
| Left inferior occipital gyrus (BA 19) | 1434 | 4.76 | <0.0001 | -44 | -84 | -4 |
| Left inferior occipital gyrus (BA 18) |  | 4.74 | <0.0001 | -38 | -84 | -12 |
| Left fusiform gyrus (BA 19) |  | 4.27 | <0.0001 | -22 | -82 | -12 |
| Right cerebellum | 2052 | 4.62 | <0.0001 | 20 | -76 | -16 |
| Right lingual gyrus (BA 18) |  | 4.49 | <0.0001 | 2 | -82 | -4 |
| Right fusiform gyrus (BA 37) |  | 4.33 | <0.0001 | 38 | -62 | -12 |
| Left cuneus (BA 18) | 350 | 4.59 | <0.0001 | -18 | -98 | 10 |
|  |  | 4.53 | <0.0001 | -8 | -104 | 12 |
| Right cerebellum | 122 | 4.50 | <0.0001 | 10 | -42 | -10 |
| Right parahippocampal gyrus (BA 30) |  | 3.59 | <0.0001 | 18 | -38 | -6 |
| Right cuneus (BA 18) | 275 | 4.49 | <0.0001 | 14 | -100 | 12 |
| Right middle occipital gyrus (BA 19) |  | 3.32 | <0.0001 | 30 | -84 | 14 |
| Left middle occipital gyrus (BA 19) | 54 | 4.11 | <0.0001 | -52 | -76 | 8 |
| Left postcentral gyrus (BA 3) | 112 | 4.00 | <0.0001 | -54 | -8 | 52 |
| *negative correlation* | | | | | | |
| Right superior occipital gyrus (BA 19) | 51 | 4.09 | <0.0001 | 48 | -78 | 30 |
| Right precuneus (BA 19) |  | 3.37 | <0.0001 | 46 | -74 | 40 |
| Left superior frontal gyrus (BA 6) | 60 | 3.42 | <0.0001 | -16 | 22 | 64 |
|  |  | 3.19 | 0.001 | -20 | 28 | 60 |

**Table S5E. Sex-specific correlations of itch intensity with brain activity during second forearm stimulation (A2) during ‘itch’ condition (uncorrected, p < 0.001, with a voxel threshold k > 47).**

| Region | k | Z-score | p | coordinates (x y z mm) | | | | |
| --- | --- | --- | --- | --- | --- | --- | --- | --- |
| **females** | | | | | | | | |
| *positive correlation* | | | | | | | | |
| Left posterior cingulate gyrus (BA 30) | 490 | 5.04 | <0.0001 | -10 | | | -60 | 16 |
| Left posterior cingulate gyrus (BA 29) |  | 4.41 | <0.0001 | -16 | | | -44 | 8 |
| Left precuneus (BA 31) |  | 4.30 | <0.0001 | -6 | | | 66 | 20 |
| Right posterior cingulate gyrus (BA 29) | 324 | 4.59 | <0.0001 | 14 | | | -42 | 14 |
| Right parahippocampal gyrus (BA 30) |  | 4.39 | <0.0001 | 20 | | | -42 | 6 |
| Right parahippocampal gyrus (BA 19) |  | 4.34 | <0.0001 | 28 | | | -46 | 0 |
| Left amygdala | 86 | 4.16 | <0.0001 | -24 | | | -10 | -16 |
| Right postcentral gyrus (BA 3) | 88 | 4.15 | <0.0001 | 52 | | | -14 | 58 |
| Right precentral gyrus (BA 4) |  | 4.00 | <0.0001 | 58 | | | -8 | 46 |
| Right cuneus (BA 18) | 147 | 4.02 | <0.0001 | 10 | | | -98 | 18 |
| Right cerebellum | 206 | 3.97 | <0.0001 | 12 | | | -78 | -10 |
| Right lingual gyrus (BA 17) |  | 3.44 | <0.0001 | 2 | | | -90 | -6 |
| Right cerebellum |  | 3.24 | 0.001 | 16 | | | -70 | 16 |
| Right superior frontal gyrus (BA 10) | 86 | 3.96 | <0.0001 | 8 | | | 60 | -8 |
| Left medial frontal gyrus (BA 10) |  | 3.81 | <0.0001 | 0 | | | 62 | -8 |
| Left cerebellum | 176 | 3.93 | <0.0001 | -22 | | | -36 | -18 |
| Left parahippocampal gyrus (BA 36) |  | 3.81 | <0.0001 | -30 | | | -32 | -14 |
|  |  | 3.44 | <0.0001 | -24 | | | -42 | -8 |
| *Negative correlation* | | | | | | | | |
| Right superior parietal lobule (BA 7) | 2529 | 4.99 | <0.0001 | 36 | | -56 | | 50 |
| Right precuneus (BA 7) |  | 4.08 | <0.0001 | 22 | | -74 | | 52 |
| Right superior parietal lobule (BA 7) |  | 3.89 | <0.0001 | 34 | | -64 | | 60 |
| Right precentral gyrus (BA 6) | 2678 | 4.92 | <0.0001 | 30 | | -2 | | 36 |
| Right middle frontal gyrus (BA 6) |  | 4.54 | <0.0001 | 42 | | 6 | | 56 |
| Right precentral gyrus (BA 9) |  | 4.29 | <0.0001 | 42 | | 4 | | 32 |
| Left middle occipital gyrus (BA 19) | 3227 | 4.66 | <0.0001 | -32 | | -86 | | 18 |
| Left cuneus (BA 18) |  | 4.62 | <0.0001 | -28 | | -100 | | 2 |
| Right cuneus (BA 18) | 618 | 4.34 | <0.0001 | 26 | | -98 | | 0 |
| Right middle occipital gyrus (BA 18) |  | 4.06 | <0.0001 | 34 | | -94 | | 2 |
| Right inferior occipital gyrus (BA 17) |  | 3.90 | <0.0001 | 26 | | -98 | | -10 |
| Left precentral gyrus (BA 6) | 368 | 4.27 | <0.0001 | -42 | | 2 | | 34 |
| Left middle frontal gyrus (BA 9) |  | 3.66 | <0.0001 | -42 | | 10 | | 32 |
| Left middle frontal gyrus BA 8) |  | 3.47 | <0.0001 | -54 | | 14 | | 38 |
| Right middle occipital gyrus (BA 19) | 72 | 3.85 | <0.0001 | 32 | | -80 | | 18 |
| Left superior frontal gyrus (BA 8) | 329 | 3.65 | <0.0001 | 0 | | 22 | | 50 |
| Right medial frontal gyrus (BA 6) |  | 3.57 | <0.0001 | 10 | | 28 | | 36 |
| Right superior frontal gyrus (BA 6) |  | 3.29 | <0.0001 | 4 | | 30 | | 60 |
| Right inferior temporal gyrus (BA 20) | 54 | 3.51 | <0.0001 | 56 | | -56 | | -14 |
| Right fusiform gyrus (BA 20) |  | 3.33 | <0.0001 | 54 | | -42 | | -22 |
| Left middle frontal gyrus; DLPFC (BA 46)* | 100 | 3.43 | <0.0001 | -44 | | 26 | | 22 |
| Left precentral gyrus (BA 9) |  | 3.24 | 0.001 | -44 | | 24 | | 34 |
| **males** | | | | | | | | |
| *positive correlation* | | | | | | | | |
| Left parahippocampal gyrus (BA 35) | 896 | 4.87 | <0.0001 | -20 | | -10 | | -24 |
| Left parahippocampal gyrus (BA 28) |  | 4.83 | <0.0001 | -22 | | -18 | | -22 |
| Left uncus (BA 36) |  | 4.18 | <0.0001 | -24 | | -2 | | -28 |
| Left precentral gyrus (BA 6) | 123 | 4.80 | <0.0001 | -16 | | -18 | | 62 |
| Left medial frontal gyrus (BA 6) |  | 3.76 | <0.0001 | -12 | -24 | | | 54 |
| Right uncus (BA 20) | 590 | 4.70 | <0.0001 | 32 | -14 | | | -26 |
| Right superior temporal gyrus (BA 38) |  | 4.30 | <0.0001 | 36 | 8 | | | -30 |
| Right uncus (BA 28) |  | 4.21 | <0.0001 | 20 | -4 | | | 24 |
| Left cuneus (BA 18) | 270 | 4.14 | <0.0001 | -4 | -100 | | | 16 |
| Right cuneus (BA 18) |  | 3.81 | <0.0001 | 16 | -92 | | | 20 |
| Left parahippocampal gyrus (BA 30) | 133 | 3.72 | <0.0001 | -14 | -48 | | | 6 |
| Left posterior cingulate gyrus (BA 30) |  | 3.70 | <0.0001 | -20 | -54 | | | 12 |
| *negative correlation* | | | | | | | | |
| Right lingual gyrus (BA 17) | 546 | 5.64 | <0.0001 | 22 | -100 | | | -8 |
| Right inferior occipital gyrus (BA 18) |  | 4.58 | <0.0001 | 38 | -88 | | | -10 |
| Right middle occipital gyrus (BA 18) |  | 4.08 | <0.0001 | 34 | -94 | | | 2 |
| Left inferior occipital gyrus (BA 17) | 1934 | 5.51 | <0.0001 | -22 | -100 | | | -14 |
| Left cerebellum |  | 5.25 | <0.0001 | -42 | -68 | | | -30 |
| Left superior parietal lobule (BA 7) |  | 5.07 | <0.0001 | -34 | -62 | | | 54 |
|  | 1131 | 5.09 | <0.0001 | -34 | -62 | | | 54 |
| Left inferior parietal lobule (BA 40) |  | 4.46 | <0.0001 | -42 | -48 | | | 56 |
| Left superior parietal lobule (BA 7) |  | 4.38 | <0.0001 | -24 | -70 | | | 56 |
| Right middle frontal gyrus (BA 9) | 2202 | 4.94 | <0.0001 | 46 | 14 | | | 32 |
| Right superior frontal gyrus; DLPFC (BA 9)* |  | 4.78 | <0.0001 | 42 | 44 | | | 34 |
| Right middle frontal gyrus; DLPFC (BA 46)* |  | 4.18 | <0.0001 | 44 | 48 | | | 22 |
| Right cerebellum | 481 | 4.85 | <0.0001 | 40 | -62 | | | -34 |
|  |  | 3.35 | <0.0001 | 40 | -74 | | | -26 |
| Right inferior parietal lobule (BA 40) | 2436 | 4.81 | <0.0001 | 40 | -48 | | | 50 |
| Right superior parietal lobule (BA 7) |  | 4.39 | <0.0001 | 36 | -62 | | | 58 |
|  |  | 4.12 | <0.0001 | 26 | -62 | | | 60 |
| Left superior frontal gyrus (BA 6) | 2525 | 4.43 | <0.0001 | 0 | 18 | | | 56 |
| Left middle frontal gyrus (BA 6) |  | 4.17 | <0.0001 | -34 | 4 | | | 62 |
| Left middle frontal gyrus (BA 9) |  | 4.14 | <0.0001 | -42 | 12 | | | 32 |
| Right inferior frontal gyrus (BA 47) | 1453 | 4.42 | <0.0001 | 46 | 18 | | | -4 |
|  |  | 4.27 | <0.0001 | 54 | 16 | | | -2 |
|  |  | 4.18 | <0.0001 | 42 | 22 | | | -12 |
| Left inferior frontal gyrus (BA 47) | 737 | 4.25 | <0.0001 | -40 | 22 | | | -10 |
| Left insula |  | 3.65 | <0.0001 | -32 | 24 | | | -2 |
| Left superior frontal gyrus (BA 9) | 389 | 4.13 | <0.0001 | -32 | 52 | | | 28 |
| Left middle frontal gyrus (BA 9) |  | 3.62 | <0.0001 | -38 | 42 | | | 34 |
| Left superior frontal gyrus (BA 10) |  | 3.47 | <0.0001 | -32 | 58 | | | 14 |
| Right middle frontal gyrus (BA 6) | 767 | 4.10 | <0.0001 | 38 | 2 | | | 48 |
|  |  | 4.06 | <0.0001 | 28 | 4 | | | 58 |
|  |  | 3.99 | <0.0001 | 36 | 6 | | | 58 |
| Left supramarginal gyrus (BA 40) | 68 | 3.75 | <0.0001 | -38 | -38 | | | 34 |

*) DLPFC = dorsolateral prefrontal cortex

**Table S5F. Sex-specific correlations of itch intensity with brain activity during second forearm stimulation (A2) during ‘Stroop’ condition (uncorrected, p < 0.001, with a voxel threshold k > 47).**

| Region | k | Z-score | p | coordinates (x y z mm) | | | | |
| --- | --- | --- | --- | --- | --- | --- | --- | --- |
| **females** | | | | | | | | |
| *positive correlation* | | | | | | | | |
| Right middle occipital gyrus (BA 18) | 1030 | 5.92 | <0.0001 | 12 | -98 | | | 14 |
| Right cuneus (BA 19) |  | 4.82 | <0.0001 | 20 | -94 | | | 24 |
| Left cuneus (BA 18) |  | 4.75 | <0.0001 | -16 | -96 | | | 20 |
| Right cerebellum | 3471 | 4.97 | <0.0001 | 46 | -64 | | | -16 |
|  |  | 4.86 | <0.0001 | 14 | -74 | | | -16 |
| Right middle occipital gyrus (BA 37) |  | 4.49 | <0.0001 | 46 | -64 | | | -6 |
| Left cerebellum | 216 | 3.85 | <0.0001 | -38 | -54 | | | -22 |
| Left fusiform gyrus (BA 37) |  | 3.68 | <0.0001 | -42 | -54 | | | -12 |
| Left cerebellum |  | 3.54 | <0.0001 | -46 | -46 | | | -26 |
| *Negative correlation* | | | | | | | | |
| Right precuneus (BA 31) | 59 | 4.56 | <0.0001 | 22 | -64 | | | 24 |
| Left precuneus (BA 31) | 52 | 3.82 | <0.0001 | -12 | -60 | | | 26 |
| **males** | | | | | | | | |
| *positive correlation* | | | | | | | | |
| Right middle occipital gyrus (BA 18) | 433 | 5.13 | <0.0001 | 16 | -100 | | | 14 |
| Right cuneus (BA 18) |  | 4.57 | <0.0001 | 22 | -92 | | | 18 |
| Right cuneus (BA 17) |  | 3.77 | <0.0001 | 26 | -80 | | | 12 |
| Right fusiform gyrus (BA 37) | 1536 | 4.75 | <0.0001 | 42 | -62 | | | -6 |
| Right cerebellum |  | 4.31 | <0.0001 | 26 | | | -62 | -22 |
| Right inferior occipital gyrus (BA 18) |  | 4.18 | <0.0001 | 28 | | | -86 | -6 |
| Left cuneus (BA 18) | 379 | 4.56 | <0.0001 | -10 | | | -102 | 4 |
|  |  | 4.37 | <0.0001 | -8 | | | -102 | 12 |
| Left middle occipital gyrus (BA 18) |  | 4.32 | <0.0001 | -18 | | | -98 | 8 |
| Left cerebellum | 583 | 4.45 | <0.0001 | -34 | | | -42 | -26 |
| Left fusiform gyrus (BA 37) |  | 3.83 | <0.0001 | -48 | | | -48 | -14 |
| Left cerebellum |  | 3.46 | <0.0001 | -34 | | | -60 | -22 |
| Left inferior occipital gyrus (BA 19) | 643 | 4.41 | <0.0001 | -40 | | | -72 | -8 |
| Left inferior occipital gyrus (BA 18) |  | 4.04 | <0.0001 | -38 | | | -82 | -10 |
| Left inferior occipital gyrus (BA 19) |  | 3.91 | <0.0001 | -40 | | | -76 | 0 |
| *negative correlation* | | | | | | | | |
| Left medial frontal gyrus (BA 6) | 297 | 4.51 | <0.0001 | -4 | | 30 | | 36 |
| Right inferior parietal lobule (BA 40) | 74 | 4.17 | <0.0001 | 66 | | -28 | | 34 |
| Right precentral gyrus (BA 44) | 82 | 3.73 | <0.0001 | 54 | | 12 | | 6 |
| Right inferior frontal gyrus (BA 47) |  | 3.48 | <0.0001 | 48 | | 14 | | 0 |
| Left inferior frontal gyrus (BA 47) | 155 | 3.71 | <0.0001 | -46 | | 22 | | -6 |
| Right superior frontal gyrus (BA 6) | 61 | 3.63 | <0.0001 | 20 | | 20 | | 60 |
| Right middle frontal gyrus (BA 10) | 78 | 3.60 | <0.0001 | 44 | | 44 | | 22 |
| Right inferior parietal lobule (BA 40) | 47 | 3.46 | <0.0001 | 56 | | -52 | | 48 |
